# Investigating Phase Separation in Genome Folding via Multiscale Computational Modeling

**DOI:** 10.1002/advs.74997

**Published:** 2026-03-28

**Authors:** Jiahu Tang, Cibo Feng, Haibin Su, Xiakun Chu

**Affiliations:** ^1^ Advanced Materials Thrust, Function Hub The Hong Kong University of Science and Technology (Guangzhou) Guangzhou Guangdong China; ^2^ Department of Chemistry The Hong Kong University of Science and Technology Kowloon Hong Kong China; ^3^ Division of Life Science The Hong Kong University of Science and Technology Kowloon Hong Kong China

**Keywords:** biomolecular condensates, data‐driven modeling, 3D genome organization, loop extrusion, polymer physics

## Abstract

The 3D organization of the genome is central to gene regulation, and phase separation has emerged as an important physical principle for this architecture. This review synthesizes how phase separation contributes to genome folding across scales, from compartmental segregation and topologically associating domains to transcriptional condensates and nucleosome arrays, with a special focus on computational advances. We organize the field into two complementary modeling paradigms: (1) physics‐based simulations, spanning all‐atom to coarse‐grained polymer representations that reveal the mechanisms driving chromatin condensation; and (2) data‐driven approaches, including machine learning, that learn structural features and regulatory interactions from high‐throughput genomic and imaging data. We highlight how integrating these models with experiments clarifies the interplay among phase separation, loop extrusion, epigenetic modifications, and the intrinsic polymer properties of chromatin in genome folding. By linking microscopic molecular interactions to mesoscale and nuclear organization, these combined approaches provide mechanistic insight into normal regulation and its dysregulation in disease, and they chart a path toward predictive, non‐equilibrium models of the 4D nucleome.

## Graphical abstract

Phase separation is emerging as a multiscale organizing principle of genome folding across scales, from nucleosomes and transcriptional condensates to chromatin domains and nuclear compartments. By integrating physics‐based simulations with data‐driven inference, computational modeling now links molecular interactions to nuclear architecture and points toward predictive, non‐equilibrium models of the 4D nucleome.

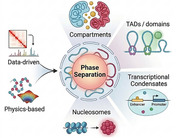

## 1 Introduction

Phase separation has emerged as a fundamental physical mechanism that organizes the spatiotemporal distribution of biomolecular components within the cell nucleus [1]. In this process, biomolecules, such as proteins, RNA, and chromatin fiber, spontaneously demix from the surrounding milieu to form dynamic, membraneless assemblies known as biomolecular condensates [2]. These condensates selectively concentrate particular molecules, thereby enhancing reaction kinetics and establishing functional microenvironments within the complex nuclear landscape. Through this capacity, condensates exert profound influence over critical biological processes, most prominently transcriptional regulation [3]. Such multifunctional roles of biomolecular condensates in genome organization, expression, and regulation have been increasingly recognized [4]. For complementary and comprehensive perspectives on multi‐scale chromatin dynamic behavior, with an emphasis on phase separation across various nuclear processes, we direct readers to several excellent recent reviews [5, 6, 7, 8].

In the context of 3D genome organization, phase separation offers a compelling physical framework for understanding chromatin architecture and dynamics [9]. Chromatin fiber, behaving as a long‐chain polyelectrolyte, possesses an intrinsic capacity for phase transitions [3, 10, 11]. This behavior is finely modulated by both the inherent properties of its constituents, such as intrinsically disordered regions (IDRs) within chromatin‐associated proteins, which drive multivalent interactions and condensate formation [2, 12], and post‐translational histone modifications that modulate chromatin's physicochemical state [13]. Extrinsic environmental factors, including energy‐consuming, non‐equilibrium processes, further shape phase behavior [14]. Crucially, the dynamical phase transformations in chromatin are strongly influenced by electrostatic forces. These arise from the polyelectrolyte nature of DNA, the positively charged chromatin‐interacting proteins (such as histones and other nuclear proteins), and the significant contributions from a multivalent mixture of mobile ions within the cellular milieu. A recent comprehensive review highlights these foundational electrostatic aspects and their central role in regulating biomolecular condensates [15]. Together, these intrinsic properties and extrinsic factors produce a spectrum of phase‐separated states, from dynamic, liquid‐like droplets to more quenched, solid‐like assemblies [2].

Deciphering the dynamics and physical underpinnings of chromatin condensates is essential for elucidating how 3D genome organization influences gene regulation and cell fate decisions.

**FIGURE 1:**
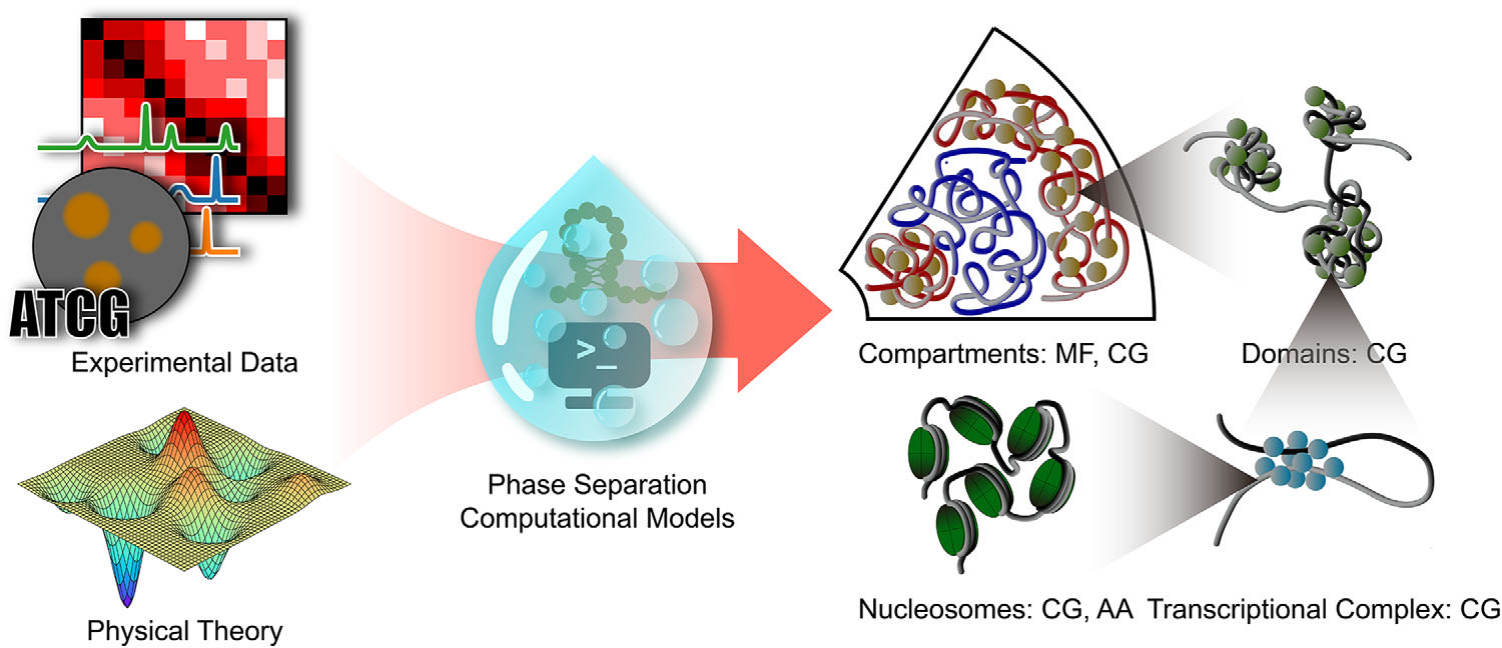
Schematic overview of multiscale genome organization and the computational frameworks that integrate experimental data with physics‐based theory. Data modalities include Hi‐C, ChIP‐seq, ATAC‐seq, imaging, and DNA sequence. Theoretical ingredients include non‐equilibrium statistical physics and effective energy landscapes. Polymer‐based computational models—which explicitly incorporate the physical interactions (such as multivalent binding and sequence‐dependent affinities) that spontaneously drive biomolecular condensation—are applied across these scales. Hierarchy of scales is detailed in: (i) Compartment scale (>1 Mb): heterochromatin (red) condenses into B compartments via protein‐driven phase separation (e.g., heterochromatin protein 1, gold), segregating euchromatin (blue) into A compartments; typical methods: mean‐field (MF) and coarse‐grained (CG) simulations. (ii) Topologically associating domains (TADs)/Domains scale (∼1 Mb): domains arise primarily through loop extrusion, with protein‐mediated phase separation (green) proposed to reinforce domain formation; typical methods: CG simulations. (iii) Gene‐regulatory scale (∼10 kb): enhancer‐promoter contacts are stabilized by transcriptional condensates composed of transcription factors and cofactors (light blue); typical methods: CG simulations. (iv) Nucleosome/fiber scale (∼200 bp): condensation of nucleosome arrays shapes local chromatin fiber; typical methods: CG and all‐atom (AA) simulations.

Genome folding is an inherently dynamic, multiscale process whose mechanistic foundations remain elusive when explored through experimental methods alone. Computational approaches have therefore become indispensable for systematically dissecting how phase separation drives the emergence of 3D genome architecture, and for elucidating the complex interplay of contributing factors. These methods enable the integration of high‐throughput and imaging datasets, such as Hi‐C [16], ChIP‐seq [17], ATAC‐seq [18], and FISH [19], with theoretical models to simulate, predict, and interpret genome behavior (Figure 1). In doing so, computational modeling not only offers mechanistic, microscopic explanations of observed phenomena but also guides the formulation of new experimental hypotheses.

Current computational strategies in the study of 3D genome organization can be neatly divided into two complementary categories: physics‐based (bottom‐up) modeling and data‐driven (top‐down) modeling [20]. Physics‐based approaches derive from fundamental laws of physics and chemistry, treating the genome as a complex physical system. Utilizing methods such as molecular dynamics (MD) and Monte Carlo (MC) simulations, these models offer mechanistic insights into how molecular interactions sculpt large‐scale genome structures and dynamics. High‐resolution all‐atom (AA) simulations capture detailed chemical interactions, making them ideal for examining the behavior of individual proteins, nucleosomes, and chromatin modifications [21, 22, 23, 24, 25, 26, 27, 28]. In contrast, coarse‐grained (CG) polymer models, which employ a “beads‐on‐a‐string” representation, enable simulations across larger genomic spans and longer timescales, facilitating exploration of mesoscale phenomena such as the formation of topologically associating domains (TADs) and compartmental segregation [29, 30, 31, 32, 33, 34, 35, 36, 37, 38, 39]. Through dynamic simulation, physics‐based models can visualize the folding of chromatin, phase‐separation behavior, and formation of biomolecular condensates, thus directly testing mechanistic hypotheses. However, because similar mesoscale phenotypes—such as chromatin compaction, clustering, and domain formation—can arise from fundamentally distinct physical mechanisms, selecting the appropriate theoretical framework is crucial. To navigate this complexity, we provide a conceptual toolbox (Box 1) that summarizes the core concepts, minimal computational ingredients, and practical diagnostics needed to distinguish classical liquid‐liquid phase separation (LLPS) from polymer‐, topology‐, and activity‐driven assemblies.

**Box 1. Conceptual Toolbox for Biomolecular Assembly in Computational Biophysics**.

Similar mesoscale phenotypes (compaction, clustering, domain formation) can arise from distinct physical mechanisms. This toolbox summarizes minimal model ingredients and practical diagnostics to distinguish classical phase separation from polymer‐, topology‐, and activity‐driven assemblies.

**Table:** 

	**LLPS**	**PPPS**	**Bridging‐induced micro‐PS**	**Percolation / Gelation**	**Activity‐driven demixing**
**Core concept**	Equilibrium demixing into dilute and dense liquid phases.	Demixing of polymers; chain connectivity, sequence, and entropy shape phase envelope.	Multivalent binders crosslink a stiff backbone (e.g., chromatin); topology frustrates macrophase separation.	System‐spanning network above a percolation threshold; elastic arrest (with or without phase separation).	Non‐equilibrium patterning maintained by energy input; detailed balance broken.
**Minimal model**	Isotropic attraction (LJ/Mie). Well depth $\varepsilon ∼k_{B}T$. Implicit solvent. Mapping via χ parameters.	Bead–spring (FENE/harmonic). Sequence‐specific (stickers/spacers). Excluded volume (WCA).	Stiff chain (WLC). Explicit multivalent binders. Topology preservation (non‐crossability).	Patchy models (valency). Deep/narrow wells ($\varepsilon \gg k_{B}T$). Explicit network/percolation analysis.	Active forces ($F_{\mathrm{act}}$), ABP/MIPS. Non‐reciprocal interactions. Time‐dependent switching.
**Morphology & Coarsening**	Full fusion; spherical droplets; macroscopic coarsening (Ostwald ripening / coalescence).	Viscoelastic fusion; delayed “necking” due to chain relaxation and entanglement.	Arrested coarsening; stable finite microdomains (“rosettes”); fusion halts at characteristic scale.	No or incomplete fusion; irregular/fractal clusters; slow shape relaxation and aging.	Dynamic steady state; size regulation, oscillations, fission‐like events; avoids simple ripening.
**Diagnostics**	Fast FRAP (Fickian diffusion); high mobile fraction; MSD linear at long times ($\Delta r^{2}\propto t$).	Delayed FRAP (viscoelastic/subdiffusive); subdiffusive MSD at short/intermediate times (α<1).	FRAP recovery set by binder $k_{\mathrm{on}}/k_{\mathrm{off}}$ rather than chain diffusion; stable morphology.	Incomplete FRAP (high immobile fraction); nonergodic; dominated by elasticity.	Energy‐dependent turnover; turning off $F_{\mathrm{act}}$ abolishes clusters; measurable entropy production.
**References**	[26, 40, 41]	[42, 43, 44]	[45, 46, 47]	[48, 49, 50]	[51, 52, 53]

*Abbreviations & Symbols*: **ABP**: Active Brownian Particle; **FENE**: Finitely Extensible Nonlinear Elastic; **FRAP**: Fluorescence Recovery After Photobleaching; **LJ**: Lennard‐Jones; **LLPS**: Liquid‐Liquid Phase Separation; **micro‐PS**: Microphase Separation; **Mie**: Mie potential; **MIPS**: Motility‐Induced Phase Separation; **MSD**: Mean Squared Displacement; **PPPS**: Polymer‐Polymer Phase Separation; S(q,t): Dynamic Structure Factor; **WCA**: Weeks‐Chandler‐Andersen; **WLC**: Worm‐like Chain.

**How to use this toolbox in practice (Simulation vs. Experiment)**

1. **Coarsening and size scaling**. Track cluster size distributions P(s) or a characteristic length scale. Continuous growth suggests LLPS/PPPS; a steady‐state plateau (not attributable to finite box size) suggests bridging‐induced frustration or activity‐stabilized microphases.
2. **Fusion and contact kinetics**. Monitor droplet coalescence: LLPS shows viscocapillary fusion and rounding; PPPS often exhibits delayed neck formation due to chain interpenetration; gels show frustrated contacts with high interfacial elasticity.
3. **Dynamics: FRAP Versus MSD**. FRAP reports exchange/mobility; simulations provide MSD and S(q,t). Subdiffusive MSD exponents (α<1) indicate polymer constraints (PPPS) or topological barriers. Plateaued FRAP and aging indicate gelation.
4. **Thermodynamic Versus active signatures**. Use “computational poisons”: reducing attractions (ε) tests equilibrium driving; turning off active terms tests non‐equilibrium dependence. Active systems typically show persistent flux and history dependence.

In contrast, data‐driven models rely on large‐scale experimental datasets to uncover chromatin structure patterns and generate predictive hypotheses. These approaches leverage machine learning and statistical methods to identify genome structural features, infer their functional relationships, and predict gene regulatory elements directly from data. For example, they can extract TADs and chromatin loops from Hi‐C contact maps or call peaks in ChIP‐seq datasets [54, 55]. Machine learning methods have also been trained to identify IDRs or assess phase‐separation propensity based solely on primary sequences [56, 57, 58]. In studies of genome architecture, data‐driven models learn mappings from 1D epigenomic features to 2D contact frequency landscapes, effectively predicting 3D chromatin interactions from sequence and epigenomic context [33, 59, 60]. Further, hybrid or inferential strategies derive effective interaction parameters from experimental data, which are then integrated into polymer simulations, thus bridging data‐driven modeling with physics‐based modeling [20, 33]. These integrative frameworks are particularly powerful for integrating multi‐omics datasets, enabling a more comprehensive understanding of how genomic variation impacts 3D genome organization.

Integrating computational modeling with experimental data has emerged as a cornerstone strategy for advancing our understanding of 3D genome folding [61, 62]. Iteratively comparing and validating model predictions against empirical observations enables systematic refinement of model hypotheses and parameters, thereby enhancing both biological fidelity and predictive power. For instance, multiscale copolymer models that incorporate sequencing and imaging data have been successfully validated against Hi‐C and super‐resolution microscopy, revealing how nanoscale heterochromatin domain formation is modulated by epigenetic reaction rates through phase separation [63]. In the following sections, we review how computational strategies, ranging from physics‐based simulations and data‐driven inferences to hybrid frameworks, have been applied to dissect the role of phase separation in genome folding across spatial and temporal scales, addressing fundamental questions of genome organization and regulation.

## 2 Phase Separation in Mesoscale Chromatin

At the mesoscale, the genome partitions into spatially segregated active (A) and inactive (B) compartments [16], a phenomenon that is now widely interpreted through the lens of phase separation, driven by heterogeneous physicochemical properties along the chromatin fiber. Polymer physics, informed by principles such as Flory‐Huggins for polymer solutions and Voorn‐Overbeek's theory for polyelectrolytes, has provided foundational frameworks for understanding macromolecular crowding and electrostatic contributions to DNA and chromatin condensation [10, 66]. Prior to the formal adoption of phase separation terminology, theoretical work already highlighted the importance of intra‐chromatin interactions and entropic forces in prompting chromosome aggregation and segregation [67, 68]. More recently, the “stickers‐and‐spacers” paradigm from polymer physics has been introduced to capture the behavior of intrinsically disordered proteins (IDPs), which are frequently associated with chromatin. Here, “stickers” provide interaction hotspots while flexible “spacers” modulate solubility and connectivity [11, 28, 42]. The physical underpinnings of these interactions remain areas of ongoing investigation [37, 69, 70]. For instance, Smrek et al. proposed that differences in non‐equilibrium activity between euchromatin and heterochromatin can drive their separation, demonstrating that for long polymers like chromatin, even a small activity differential is sufficient to induce demixing [71]. Conceptual advances have also delineated two interrelated modes of chromatin phase behavior: (i) Polymer‐polymer phase separation (PPPS), which is driven by chromatin cross‐linking via bridging factors [3], and (ii) LLPS, which is facilitated by multivalent interactions among soluble proteins that selectively incorporate or exclude chromatin segments [72].

**FIGURE 2:**
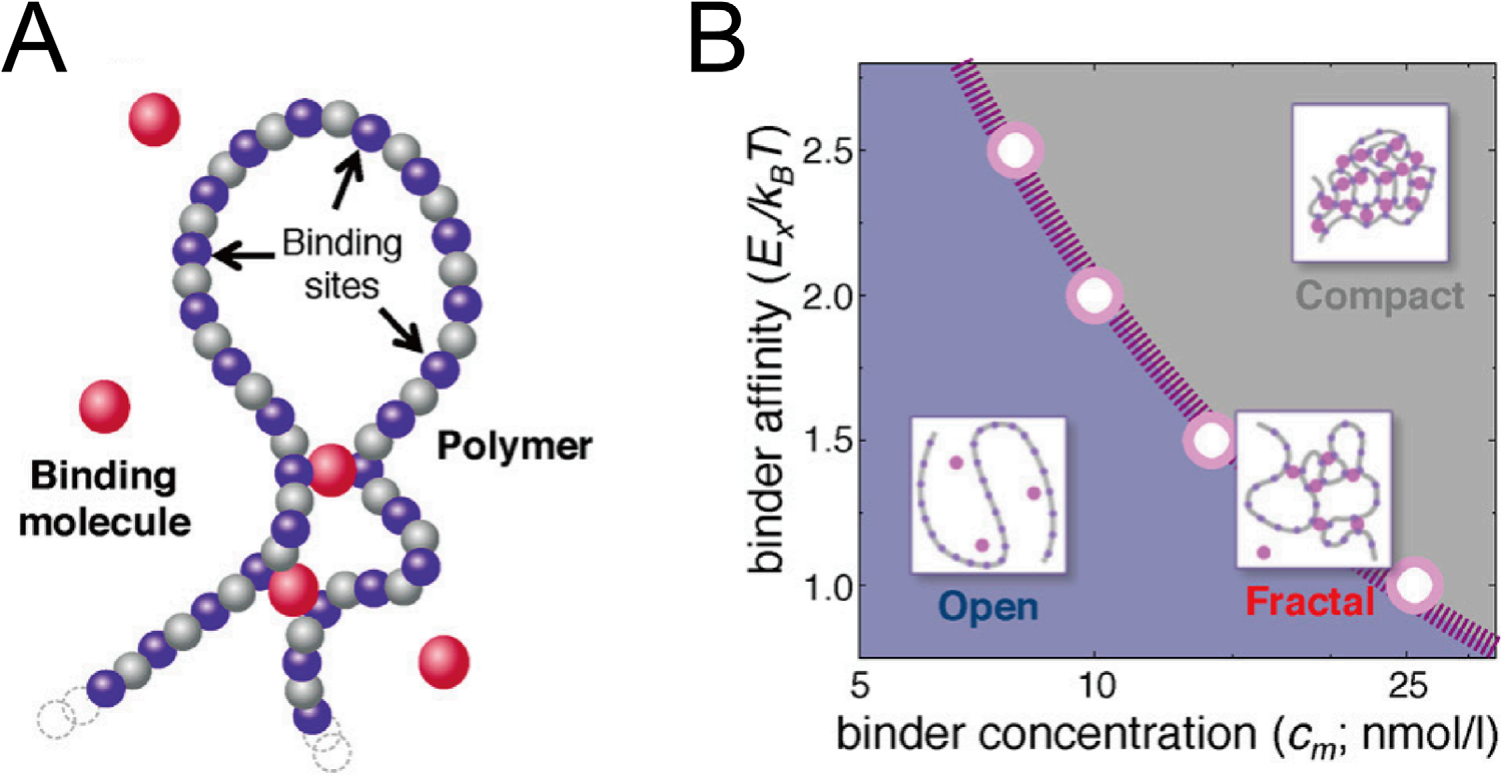
Schematic representation of the chromatin‐assisted protein condensation model based on the “strings‐and‐binders switch” (SBS) framework [64]. (A) The model comprises a self‐avoiding polymer (chromatin) depicted as a chain of beads, some of which bear specific binding sites (blue spheres) with diffusing beads (chromatin‐associated proteins, magenta spheres) while others are non‐binding (gray spheres). Diffusing binder molecules interact either specifically or nonspecifically with chromatin beads. (B) A phase diagram illustrates the conformational regimes of the model as a function of binder concentration and binding affinity: a weak‐interaction open state with randomly folded chromatin; a compact, highly folded state above the critical threshold; and a fractal‐like intermediate structure at the transition point. (Adapted from Barbieri et al., *Proc. Natl. Acad. Sci. U.S.A*. 109, 16173‐16178, 2012. [64]).

A more concrete depiction involves the chromatin‐assisted condensation of specific proteins, a framework that explicitly models both the chromatin polymer and phase‐separating proteins (Figure 2). Sommer et al. introduced a mean‐field (MF) model, termed polymer‐assisted condensation (PAC), and corroborated it through MD simulations, revealing that chromatin can nucleate condensates even when binder concentrations remain well below the critical bulk threshold [73, 74]. Complementing this, Tortora et al. used a minimal theoretical framework to demonstrate that heterochromatin protein 1 (HP1) can form stable condensates at concentrations lower than those required for in vitro phase separation, emphasizing the role of chromatin in lowering the nucleation barrier [75]. These studies underscore a bidirectional interplay in which chromatin compaction and protein phase separation reinforce each other [76]. Tortora et al. further showed that continuous tracts of H3K9me2/3 facilitate rapid condensation, whereas sparse or dynamically established methylation landscapes yield sluggish, viscoelastic kinetics, explaining the persistence of multiple small nuclear condensates, such as nucleoli, instead of their fusion into a single large body [44, 60]. Additionally, chromatin‐assisted condensation models capture varied nucleation processes, including chromatin‐independent, chromatin‐templated, and purely stochastic regimes, potentially corresponding to the formation of distinct nuclear bodies [77]. In contrast, alternative models propose that binders associating with active euchromatin improve its solvent quality, causing it to swell and effectively compact adjacent heterochromatin into core‐shell assemblies, as directly visualized in live‐cell imaging studies [78, 79].

**FIGURE 3:**
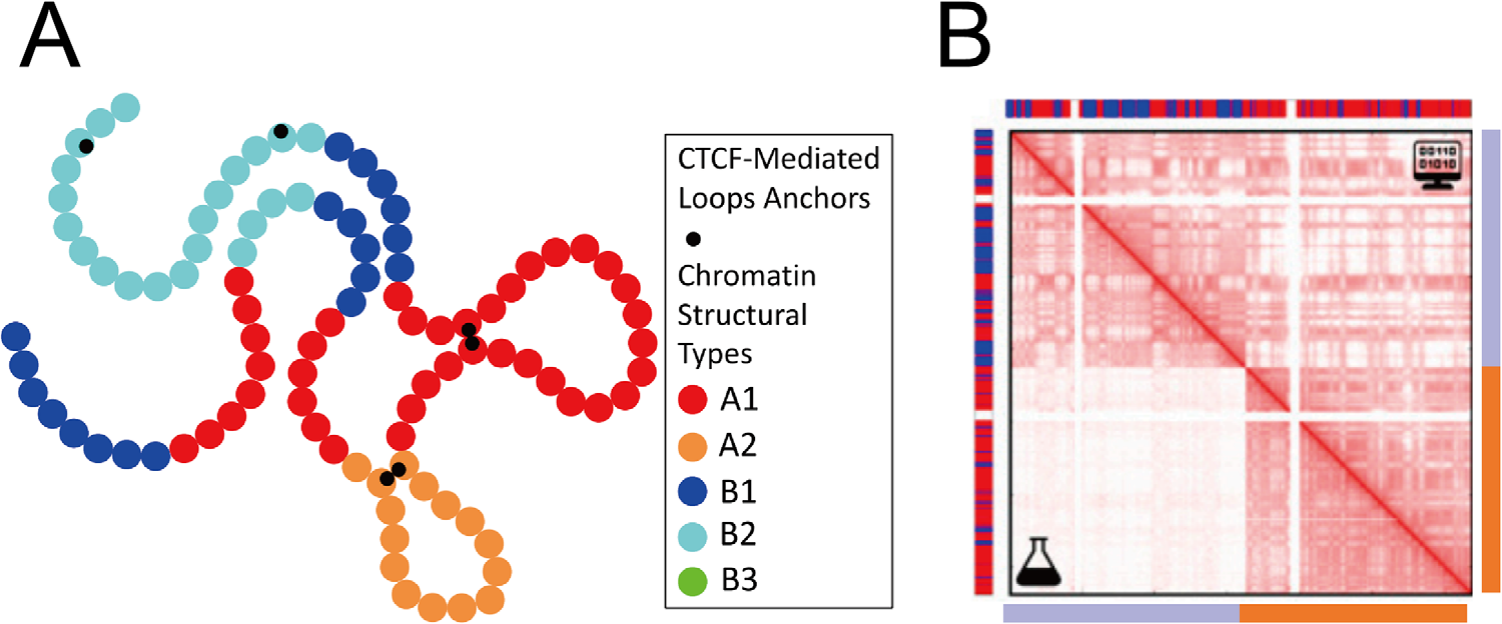
Block copolymer model for chromosome folding, illustrating how epigenetic state informs 3D genome interactions [65]. (A) The chromatin polymer is represented as a self‐avoiding “beads‐on‐a‐string” chain, where each bead corresponds to a chromosomal segment (e.g., 40 kb) and is colored according to its epigenetic classification. Beads of different types interact via type‐dependent affinities, reflecting epigenetically mediated attractions. (B) When only epigenetic annotations (bars at top and alongside the heatmap) are used as input, this model successfully reproduces characteristic patterns of chromosomal contacts: the lower‐left triangle depicts the experimental Hi‐C contact frequency, while the upper‐right triangle shows the simulated contact map generated by the computational model. (Adapted from Di Pierro et al., *Proc. Natl. Acad. Sci. U.S.A*. 115, 7753‐7758, 2018 [65]; licensed under CC BY‐NC‐ND 4.0).

Phase separation offers a framework for translating 1D epigenetic information into 3D genome architecture, as exemplified in both physics‐based models such as the “strings‐and‐binders switch” (SBS) [64, 81, 82] (Figure 2) and data‐driven models such as MiChroM [21, 65, 83, 84, 85, 86] (Figure 3). In the SBS framework, chromatin is modeled as a polymer with binding‐active and inert beads, together with explicit protein binders that interact with chromatin via type‐specific affinities (Figure 2A), generating phase behavior dependent on binder concentration and interaction strength (Figure 2B). MiChroM, by contrast, employs a block copolymer approach: protein affinities are implicitly embedded within chromatin‐chromatin interactions based on epigenetic block identities (Figure 3A‐B).

**FIGURE 4:**
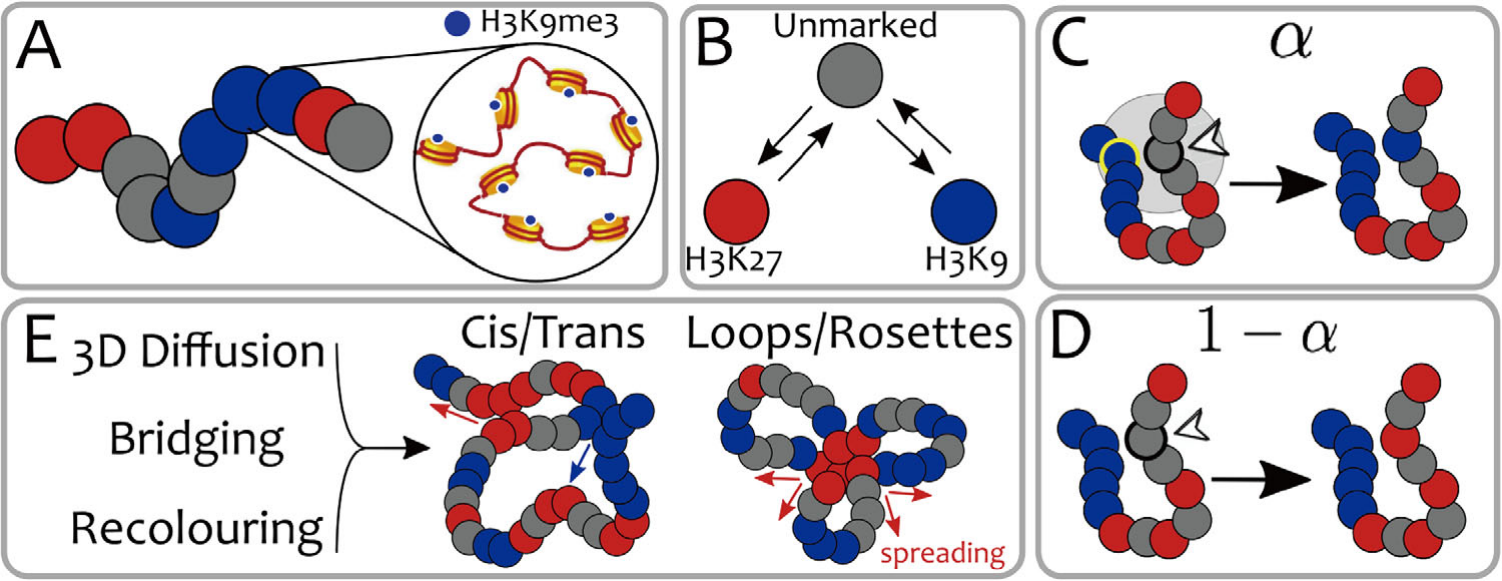
The “genomic bookmarking” model, illustrating the coupling between chromatin structure and epigenetic state dynamics [80]. (A) Chromatin is represented as a polymer chain where each bead is colored to reflect its current epigenetic mark. (B) Beads may change color over time in response to changing biophysical conditions (e.g., activity of writer and eraser enzymes). (C) At each recoloring event, a bead has probability α of adopting the epigenetic color of a randomly selected 3D‐proximal neighbor (highlighted in yellow). (D) Alternatively, with probability 1−α, the bead changes to a different epigenetic state than that of the neighbor. (E) The interplay of 3D chromatin dynamics, bridging interactions (by reader proteins), and local epigenetic recoloring creates dynamic genome architectures and promotes spreading of epigenetic domains in both cis and trans. (Adapted from Michieletto et al., *Nucleic Acids Res*. 46, 83‐93, 2018 [80]; licensed under CC BY‐NC 4.0).

Far from static, the epigenetic landscape is a dynamic, adaptive layer that both reshapes and is reshaped by 3D genome architecture [87, 88, 89, 90, 91, 92] (Figure 4). Non‐equilibrium “reader‐writer” polymer models in which marks are continuously written and erased reproduce the coexisting micro‐phase separation observed in cells, whereas equilibrium formulations tend to over‐segregate [93]. These feedback‐driven systems are highly sensitive: modest changes in reader or writer activities can trigger large, switch‐like shifts between chromatin states, offering routes for differentiation that are themselves modulated by chromatin activity [94, 95, 96, 97, 98, 99, 100, 101, 102]. The coupling is bidirectional as 3D proximity biases the writing of epigenetic marks, which in turn remodels folding, yielding transitions between mixed (disordered) and phase‐separated (ordered) states in theory and simulation [39, 103, 104]. To account for memory across cell cycles, “genomic bookmarks”, i.e., permanently marked, rewrite‐resistant loci, have been proposed, where models predict a critical bookmark density below which the system collapses to a single epigenetic state via a first‐order transition [80, 105]. Extensions that allow marks to spread to 3D‐proximal neighbors further recapitulate the emergence and maintenance of heterochromatin domains and their boundaries in agreement with experiments [63].

Beyond protein‐mediated interactions, the intrinsic 1D DNA sequence can also encode a propensity for large‐scale genome segregation. Liu et al. developed a model based on CpG island (CGI) density, classifying the genome into CGI‐rich “forests” (F) and CGI‐poor “prairies” (P) [106]. They found these regions possess distinct genetic and epigenetic properties and tend to spatially segregate, providing a unifying framework to describe changes in genome compartmentalization during cell development, differentiation, senescence, and cancer. The authors further proposed that the hydrophobicity of prairies promotes F/P segregation but inhibits inter‐chromosome territorialization. Notably, TAD lengths appear evolutionarily tuned to minimize chromosomal phase separation while preserving territorial integrity, aligning with forest/prairie domain scales [107]. Moreover, varying loop types are believed to differentially modulate phase separation dynamics [108].

**FIGURE 5:**
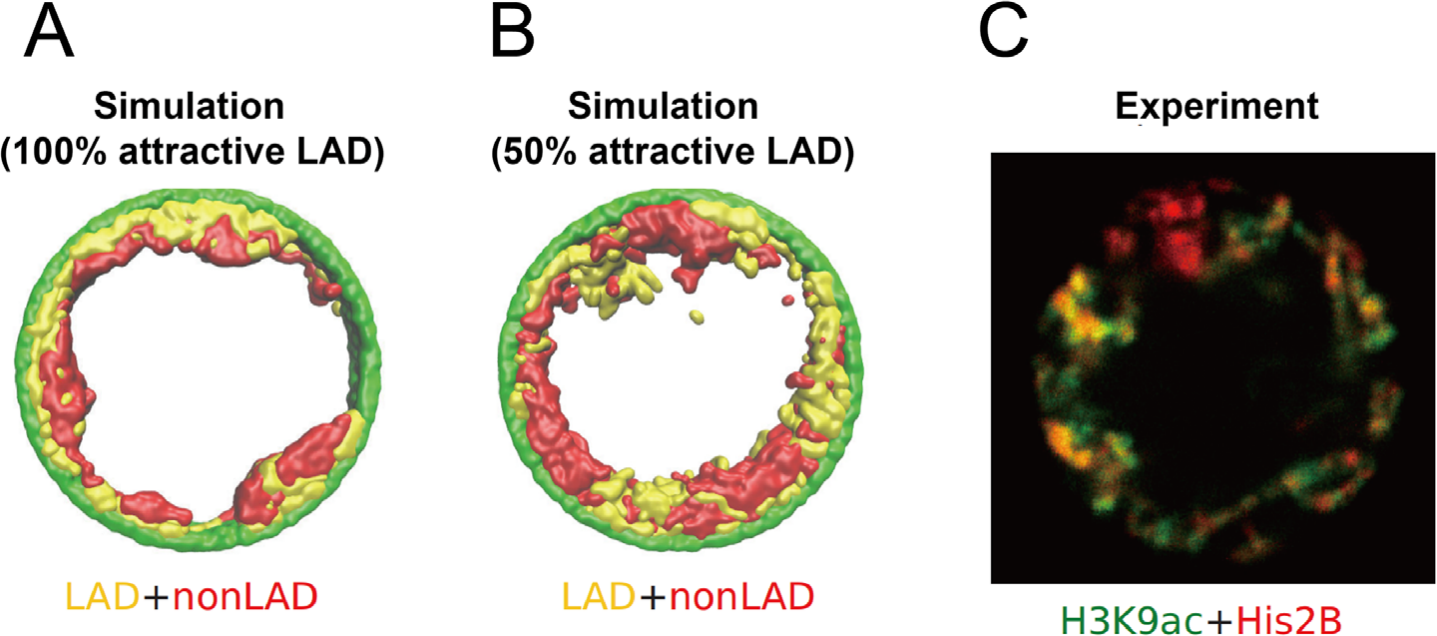
Nuclear lamina‐mediated compartmentalization of chromatin, illustrating how varying fractions of lamina‐associated domains (LADs) influence mesoscale genome organization [109]. (A) When 100% of chromatin comprises LADs that bind the nuclear lamina, chromatin concentrates peripherally in a continuous radial shell, demarcating clear separation between LAD and non‐LAD regions. (B) With approximately 50% LAD content, simulations predict angular segregation on the nuclear periphery, yielding alternating patches of LAD and non‐LAD chromatin rather than uniform radial layering. (C) Experimental imaging data in live Drosophila muscle nuclei reveal analogous angular partitioning, in line with the theoretical prediction. (Adapted from Bajpai et al., *eLife* 10, e63976, 2021 [109]; licensed under CC BY‐NC 4.0).

The global organization of compartments is critically constrained by the nuclear environment, most notably the nuclear lamina. Bajpai et al. demonstrated via CG polymer simulations that by tuning three key parameters, i.e., chromatin self‐attraction, lamina binding affinity, and chromatin volume fraction, they could reproduce four distinct, experimentally observed nuclear organization modes: conventional, peripheral, central, and wetting droplet configurations [109]. Intriguingly, their simulations predicted that under certain conditions, lamina‐associated domains (LADs) and non‐LAD regions could micro‐phase‐separate in an angular direction along the periphery, rather than radially (Figure 5). The central role of the lamina in nuclear organization is further underscored by Martin et al., who showed that abolishing lamina‐chromatin interactions yields an “inverted” nuclear architecture, with heterochromatin aggregating at the nuclear center [110]. Complementary phenomenological models, such as field‐theoretic approaches, have also been utilized to characterize the interactions among chromatin, the lamina, and the surrounding nucleoplasm [111, 112, 113]. Moreover, lamina association modulates other phase separation‐mediated chromatin structures: for example it suppresses centromere self‐aggregation [114], and conversely, chromatin phase behavior can influence nuclear morphology and fluctuation dynamics, which are often dysregulated in disease states [35]. Beyond the lamina, prominent nuclear bodies like the nucleolus also form via phase separation, driven by self‐organization of rDNA loci. Polymer‐based modeling has shown that nucleolar condensation is robust to the genomic location of rDNA, highlighting phase separation as a general organizing principle of nuclear mesoscale structures [115]. Functionally, the mesoscale phase separation of these large genomic compartments is vital for maintaining cellular identity and genomic stability [16, 116]. For instance, the spatial sequestration of heterochromatin at the nuclear periphery represses lineage‐inappropriate genes during differentiation [117] and prevents aberrant recombination between repetitive DNA elements [118]. Dysregulation of these macro‐scale condensates is a hallmark of cellular senescence and various cancers, where the loss of compartmental boundaries leads to widespread epigenetic dysregulation and altered gene expression profiles [119].

## 3 Phase Separation in Chromatin Domains

Within the large‐scale A/B compartments, the genome is further organized into TADs, which appear as characteristic square‐like domains on Hi‐C contact maps [54, 120]. These domains are primarily established by the ATP‐dependent process of loop extrusion [121, 122, 123, 124, 125], where cohesin extrudes chromatin loops until stalled by boundary elements, often marked by convergently oriented CTCF proteins [126]. Functionally, TADs act as insulated regulatory units that confine enhancer‐promoter interactions, thereby ensuring transcriptional specificity and contributing to a knot‐free chromatin conformation [9, 127]. Disruption of TAD boundaries can lead to aberrant regulatory events, such as ectopic enhancer activation, which is implicated in developmental disorders and cancer [128].

While loop extrusion provides the primary framework for TAD formation, there is mounting evidence that thermodynamic principles, particularly phase separation, play a crucial, synergistic role [31, 36, 129]. For instance, the reconstruction of TADs during cellular reprogramming has been shown to be regulated by OCT4‐driven phase separation [130]. Polymer‐physics simulations have been essential for dissecting the interplay between loop extrusion and phase separation. By explicitly encoding extruder dynamics and multivalent binding/segregation, these models enable direct tests of mechanistic hypotheses and quantify how competing and cooperative forces sculpt TAD architecture [120, 131]. A key insight from these models is the concept of state degeneracy, which proposes that a phase‐separated chromatin domain is not a single, static structure but rather a thermodynamically favorable ensemble of many distinct, yet related, conformations [64]. This inherent variability successfully explains the extensive cell‐to‐cell heterogeneity observed in single‐cell experiments, while the population average of this ensemble reproduces the well‐defined structures seen in bulk Hi‐C data [83, 132].

**FIGURE 6:**
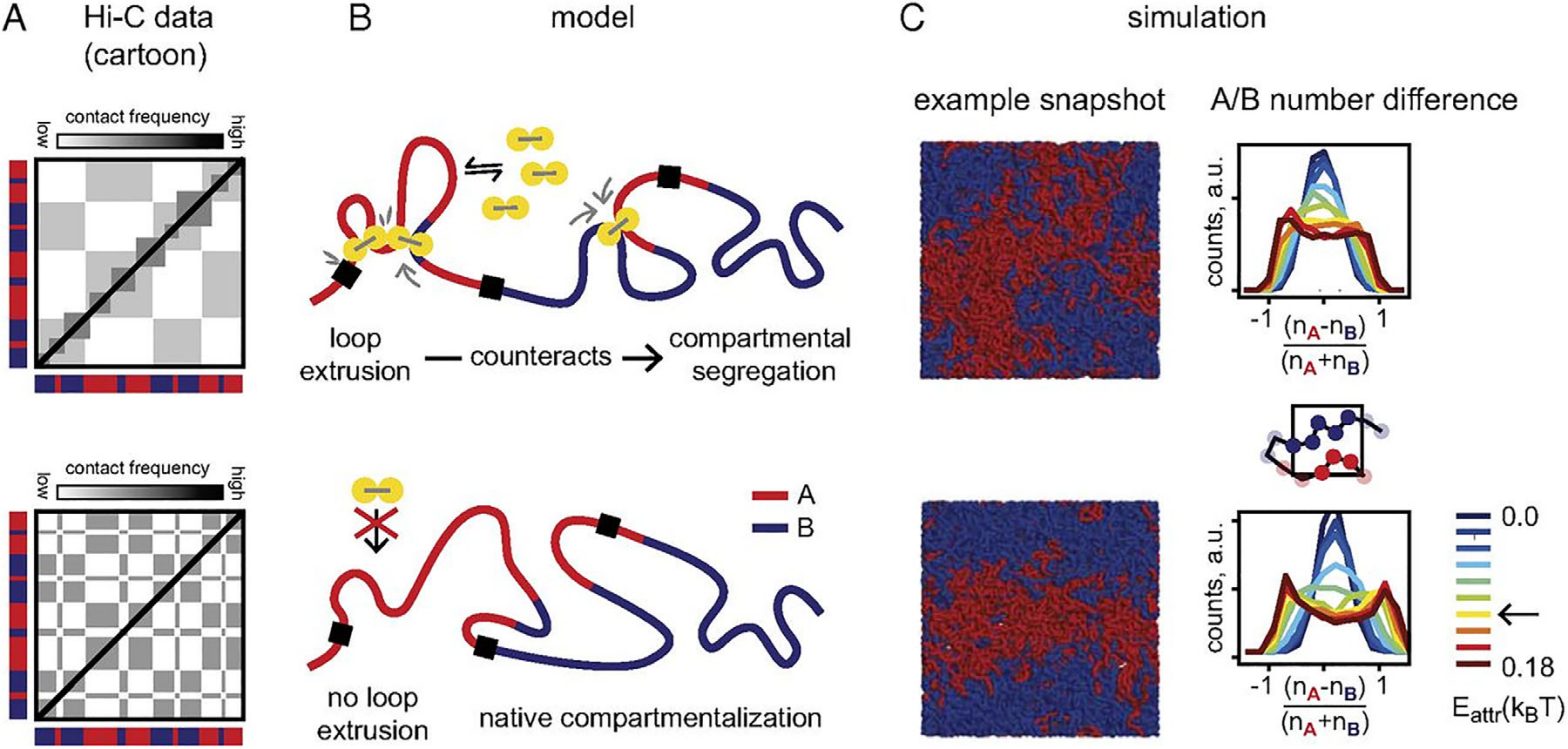
Interplay between loop extrusion and phase separation in shaping 3D genome architecture, as revealed by polymer modeling [116]. (A) Experimental Hi‐C data show that removal of cohesin abolishes TADs while enhancing compartmentalization, highlighting an antagonistic relationship between local loop formation and global compartment segregation. (B) The polymer model posits that active loop extrusion (mediated by cohesin) promotes mixing of chromatin domains, counteracting phase separation driven by differential affinities between A‐ and B‐type chromatin. (C) Simulation results recapitulate experimental observations: loss of loop extrusion enhances compartmental domain formation, while increased cohesin processivity strengthens TADs but weakens compartmental segregation. These findings underscore that genome organization is a dynamic, non‐equilibrium balance between active mixing and passive compartmental segregation mechanisms. (Adapted from Nuebler et al., *Proc. Natl. Acad. Sci. U.S.A*. 115, E6697‐E6706, 2018 [116]; licensed under CC BY‐NC‐ND 4.0).

Importantly, computational models show that loop extrusion and phase separation are not mutually exclusive but exist in dynamic competition within the genome [36, 133, 134, 135, 136]. The efficacy of this competition hinges on the processivity and mechanical stability of the extrusion machinery. Complementing mesoscale polymer studies, multiscale computational approaches have recently zoomed in on the molecular mechanics of the motor itself. Recent work revealed that DNA actively regulates the “safety‐belt” dynamics of condensin, acting as a structural latch that ensures stable entrapment during loop expansion [137]. Such molecular‐level stability provides the mechanistic basis for loop extrusion to act as a robust non‐equilibrium mixing force, which, as shown by Nuebler et al., counteracts thermodynamically driven compartmental segregation (Figure 6) [116]. They found that active loop extrusion acts as a non‐equilibrium mixing force that counteracts thermodynamically driven compartmental segregation, especially at length scales smaller than the processivity of cohesin. This explains why cohesin depletion strengthens finer‐scale compartments by revealing the intrinsic segregation patterns of genome. This model captures the trade‐off between TAD formation and compartmental segregation, reflecting how these structures are tuned by perturbations to loop extrusion activity. Moreover, models that include both explicit binders and their non‐equilibrium binding‐unbinding kinetics are essential for quantitative agreement with experimental observations and for preventing unbounded condensate growth [138, 139]. Supporting the idea of coexistence, a recent polymer modeling study compared loop‐extrusion‐only, phase‐separation‐only, and combined hybrid (loop extrusion and phase separation) frameworks [36]. This study revealed that single‐cell chromatin conformations, especially higher‐order contacts, are best captured by the hybrid model, providing strong evidence that loop extrusion and phase separation operate in synergy even at the single‐molecule level. Additional modeling predicts novel phases, such as compact, disordered chromatin emerging from nonspecific binder contributions, and further suggests that loop formation itself may drive A/B compartmentalization through explosive percolation‐like transitions [140, 141]. Together, these findings suggest a hierarchical interplay: loop extrusion defines TAD‐like structures by actively mixing chromatin, while phase separation refines fine‐grained, functional condensates within these dynamically established contexts [31]. Thus, loop extrusion and phase separation jointly sculpt genome architecture, with loop extrusion delineating structural domains and phase separation tuning their internal organization.

**FIGURE 7:**
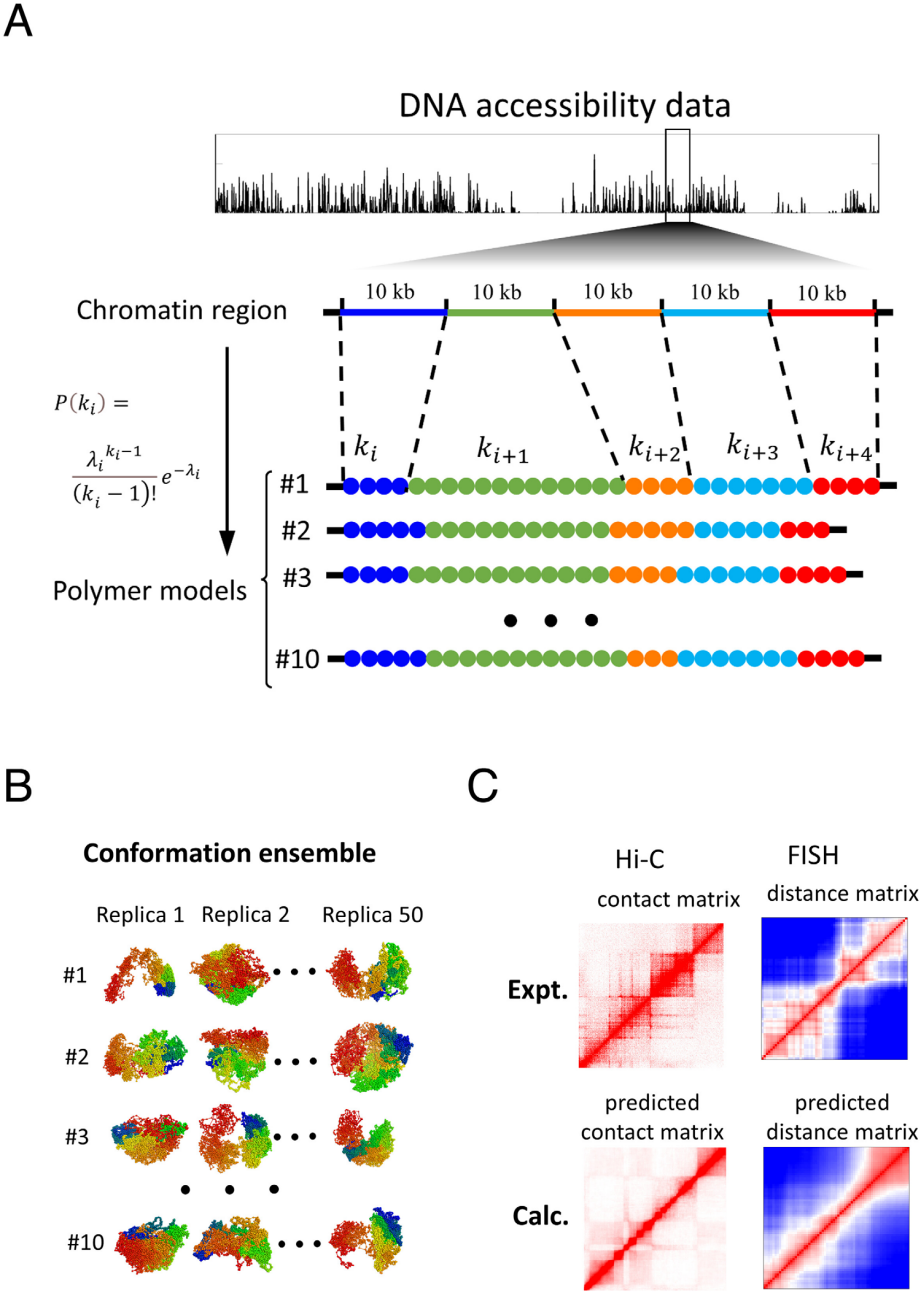
Computational workflow linking chromatin DNA‐packing density to TAD formation via polymer modeling [120]. (A) Chromatin accessibility data, for instance from ATAC‐seq or DNase‐seq, is used as a proxy for local DNA‐packing density. This information parameterizes a heteropolymer model: each genomic segment is represented by a bead whose packing density reflects its measured accessibility. (B) Stochastic simulations of these heteropolymers, which reflect the 3D folding of chromatin in a confined nuclear volume, generate an ensemble of possible conformations. (C) From this ensemble, simulated contact maps and spatial‐distance matrices are derived and compared against population Hi‐C and FISH experiments, respectively. The model successfully captures over 60% of empirically observed TAD boundaries and recapitulates spatial organization of chromatin loci. (Adapted from Meng et al., *Proc. Natl. Acad. Sci. U.S.A*. 122, e2418456122, 2025 [120]; licensed under CC BY‐NC‐ND 4.0).

The formation of TAD boundaries, critical for their insulating function, also arises from multiple factors. In the loop extrusion model, boundaries are anchored by convergently oriented CTCF binding sites [120] and are enriched with active epigenetic marks, indicating that active chromatin states contribute to their demarcation [142]. Beyond these molecular anchors, computational studies have highlighted a distinct physical principle: heterogeneous DNA packing density along the chromatin fiber. Regions of lower packing density can act as physical barriers, promoting segregation between neighboring domains [120]. For example, the computational workflow developed by Meng et al., demonstrates how chromatin accessibility data can be used as a proxy for packing density to successfully predict 3D genome organization and validate it against experimental data (Figure 7) [120]. Polymer physics models are thus essential for exploring this complex landscape, which is shaped by the interplay of sequence‐specific factors, active enzymatic processes, and fundamental physical forces [34, 143].

## 4 Phase Separation in Transcriptional Condensates

Phase separation not only participates in the large‐scale chromatin compartmentalization and TAD formation, but also fine‐tunes gene expression by forming transcriptional condensates. These are membraneless organelles formed via phase separation, functioning as hubs that concentrate essential machinery, including RNA polymerase II (Pol II), transcription factors, and coactivators such as Mediator and BRD4, at specific genomic loci (e.g., enhancers) [144, 145, 146, 147]. Formation of these condensates is driven by weak, multivalent interactions, primarily among the IDRs of regulatory proteins, which elevate the local concentration and residence time of transcriptional machinery, thereby enhancing the efficiency of transcription initiation [148, 149, 150]. The biophysical properties of these condensates are finely tuned; for instance, phosphorylation of the RNA Pol II C‐terminal domain (CTD) can alter its phase behavior, switching condensates from a state of initiation to one that recruits splicing factors, thus integrating multiple steps of gene expression [151, 152].

A central question in transcriptional regulation is how multivalent molecular interactions confer distinctive functional properties to condensates, especially the hypersensitivity of super‐enhancers (SEs). Conceptual models by Hnisz et al. propose that transcriptional regulators act as multivalent units, and their stochastic simulations demonstrate that systems with many interacting components (e.g., SEs) undergo sharp, switch‐like (ultrasensitive) phase transitions at lower interaction thresholds than simpler systems, such as typical enhancers [147]. This emergent “ultrasensitivity” provides a physical mechanism for the observed vulnerability of SEs to perturbations and their ability to drive robust transcriptional bursting [147]. Beyond conceptual frameworks, CG MD simulations enable exploration of condensate formation dynamics and material properties, such as viscosity and surface tension, which in turn influence internal reaction kinetics [153]. For example, Alshareedah et al. showed that condensates formed from multivalent protein‐RNA mixtures exhibit tunable viscoelastic behavior. These condensates behave like Maxwell fluids, which are elastic on short timescales and fluid‐like at longer durations, with alpha relaxation times and viscous versus elastic dominance modulated by sticker‐spacer sequence features and component ratios [148]. These findings underscore that the kinetics and mechanics of condensate formation cannot be inferred solely from equilibrium thermodynamics; instead, both thermodynamic drivers and kinetic constraints shape their behavior and function [149, 154].

**FIGURE 8:**
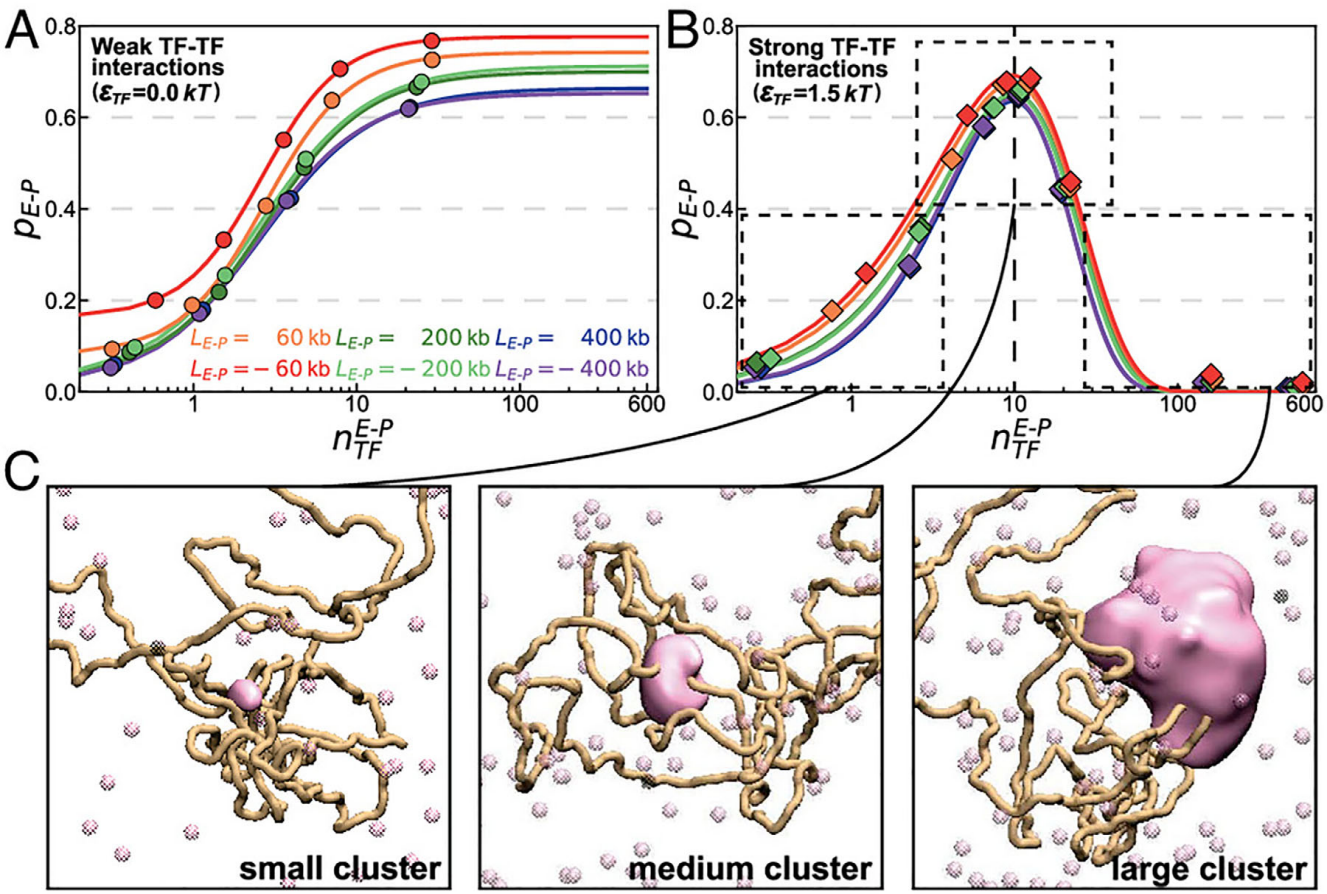
The “golden mean” principle governing enhancer–promoter (E‐P) communication within transcriptional condensates [156]. (A) Under weak TF‐TF interactions, the E‐P contact probability increases monotonically with the number of TFs in the cluster. (B) Stronger TF interactions lead to a non‐monotonic (bell‐shaped) relationship, where E‐P communication peaks at an intermediate condensate size. (C) Representative simulation snapshots illustrating that both insufficient clustering and excessive condensation (leading to molecular crowding) reduce E‐P contact efficiency. (Adapted from Zhu et al., *Proc. Natl. Acad. Sci. U.S.A*. 122, e2513371122, 2025 [156]; licensed under CC BY‐NC‐ND 4.0).

Functionally, transcriptional condensates act as phase‐separated hubs that significantly influence 3D genome architecture by facilitating long‐range chromatin interactions, effectively bridging distal enhancers and their target promoters [127, 145, 155, 156]. Polymer physics models have been particularly insightful for dissecting this phenomenon. For example, the SBS framework and related polymer simulations show that assigning homotypic “sticky” interactions to beads representing regulatory elements enables the spontaneous formation of interaction domains. These simple physical rules are sufficient to reproduce complex patterns observed in Hi‐C datasets, underscoring how multivalent interactions among regulatory proteins can drive large‐scale genome organization through phase separation‐like behavior [110, 127]. However, higher‐order condensation does not always translate to increased functional interactions. Our recent work using polymer simulations identifies a “golden mean” principle governing this process: enhancer‐promoter contact frequency peaks at intermediate levels of transcription factor (TF) clustering (Figure 8). This suggests that while initial phase separation facilitates bridging, excessive condensation may paradoxically impair communication due to molecular crowding and competing effects [156]. Such a biphasic response highlights the need for precise homeostatic control of protein concentrations within transcriptional hubs.

**FIGURE 9:**
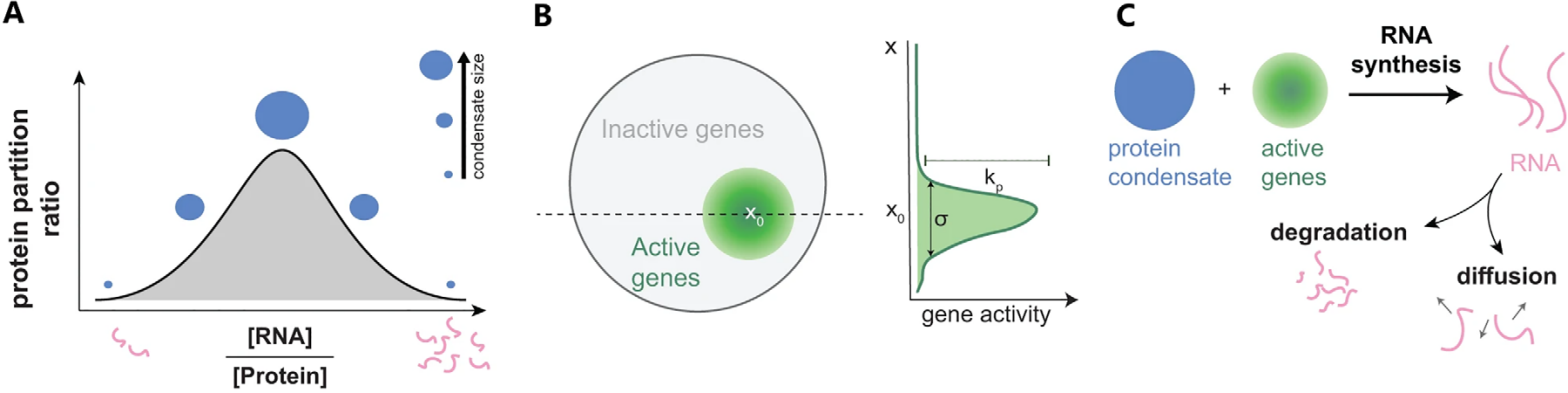
Physics‐based model depicting how gene activity and spatial gene clustering govern nuclear condensate formation and dynamics [157]. (A) The model integrates three core principles: (i) re‐entrant phase behavior of RNA‐protein mixtures, where condensate formation is maximized at optimal RNA‐to‐protein ratios; (ii) spatial clustering of active genes, represented as localized regions of heightened transcription activity; and (iii) non‐equilibrium transcription dynamics, including RNA synthesis, diffusion, and degradation. (B) When transcriptional activity is low or moderately clustered, localized gene clusters nucleate condensates via positive feedback between RNA production and protein recruitment. (C) At high transcription rates and strong clustering, condensates adopt aspherical or irregular shapes, migrate toward distant gene clusters following RNA gradients, and exhibit activity‐dependent repositioning and competition between clusters. Together, these features yield a unified framework that predicts how non‐equilibrium gene activity and genome organization shape diverse condensate morphologies and dynamics. (Adapted from Schede et al., *Nat. Commun*. 14, 4152, 2023 [157]; licensed under CC BY 4.0).

**FIGURE 10:**
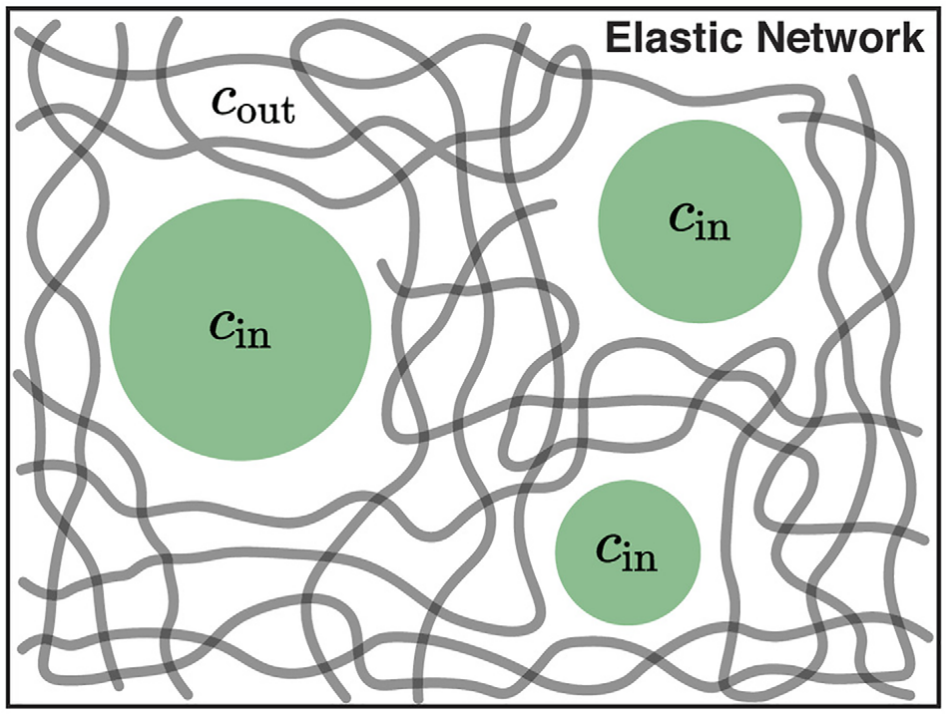
MF model illustrating condensate dynamics within a heterogeneous elastic chromatin‐like medium [158]. Biomolecular condensates (with fixed internal concentration $C_{\text{in}}$) emerge within a shared dilute phase ($C_{\text{out}}$). Each condensate is embedded in an elastic network exerting a distinct local pressure that reflects chromatin mechanical heterogeneity. This mechanical heterogeneity drives a unique form of “elastic ripening”, distinct from classical Ostwald ripening, where condensate growth dynamics follow a temporal power‐law scaling dependent on the local stiffness distribution. By incorporating an active dissolution mechanism, which describes the effects of RNA accumulation, the model reproduces three classes of condensate kinetic behaviors and reveals that burst frequency decays exponentially with local stiffness, thereby linking mechanical properties of chromatin to transcriptional bursting kinetics. (Adapted from Meng et al., *Proc. Natl. Acad. Sci. U.S.A*. 121, e2316610121, 2024 [158]; licensed under CC BY‐NC‐ND 4.0).

Crucially, transcription is an active, non‐equilibrium process, so transcriptional condensates are inherently dynamic and shaped by ongoing cellular activity. Recent models now incorporate energy‐dependent reactions and mechanochemical feedback into their frameworks. One such model by Schede et al. explores how spatial clustering of active genes, combined with re‐entrant RNA‐protein phase behavior, creates feedback loops in condensate dynamics: nascent RNA synthesis can locally alter condensate composition and stability, enabling condensates to nucleate, grow, reposition, or dissolve in response to gene activity (Figure 9) [157]. In parallel, a complementary model by Meng et al. embeds condensates within a heterogeneous elastic medium mimicking chromatin mechanics (Figure 10); it demonstrates that spatial variations in stiffness impose distinct elastic pressures on condensates, resulting in a novel “elastic ripening” phenomenon that modulates the kinetics of transcriptional bursting [158]. Together, these models reveal that transcriptional condensate behavior emerges from a rich interplay between molecular interactions, active RNA synthesis and degradation, and the mechanical properties of the nuclear environment. This integrated view unites non‐equilibrium biochemical feedback with physically grounded mechanochemical regulation in shaping nuclear condensates.

While these models offer powerful insights, confirming the existence of transcriptional hubs driven by many‐body interactions from population‐averaged Hi‐C data remains challenging. A promising solution is the computational tool CHROMATIX, which deconvolves Hi‐C contact matrices to reconstruct ensembles of single‐cell chromatin conformations and uncover multi‐way interactions otherwise obscured in aggregated data [159]. With applications to SE regions, CHROMATIX revealed significant many‐body contacts that suggest cohesive transcriptional hub formation. These computationally inferred hubs are further supported by colocalization with active RNA Pol II and Polycomb components, reinforcing a phase‐separation‐based model of functional interaction domains.

Beyond driving steady‐state transcription, the dynamic nature of these condensates allows cells to rapidly integrate environmental signals and orchestrate cell fate transitions. In pathological contexts, understanding such phase‐separated structures has profound implications for health and disease. For example, the aberrant phase separation of chimeric fusion proteins (such as EWS‐FLI1 in Ewing sarcoma or NUP98 fusions in leukemia) creates oncogenic super‐enhancers that hijack normal gene expression programs [160]. Computational methods can predict how cancer‐associated mutations within the IDRs of transcription factors may alter phase separation behaviors and remodel interaction networks, shedding light on disease mechanisms and aiding therapeutic strategy development [110]. As phase separation plays a pivotal role in oncogenic chromatin regulation, modulating condensate biophysical properties —such as dissolving pathological hubs while preserving physiological ones—becomes a promising avenue for therapeutic intervention [161].

## 5 Phase Separation in Nucleosomes, Nucleosome Arrays, and Associated Proteins

**FIGURE 11:**
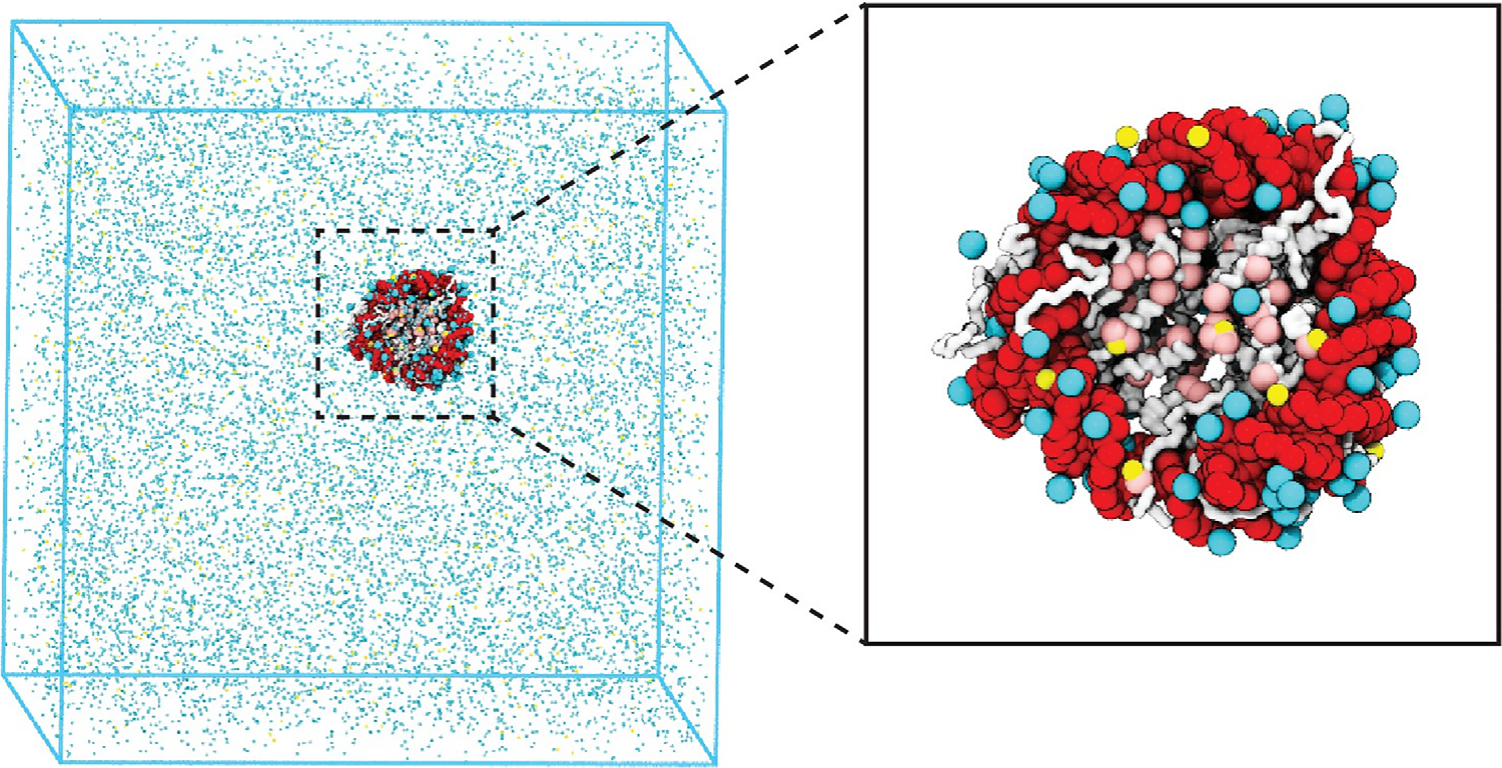
Physics‐based explicit‐ion modeling quantifies intrinsic nucleosome‐nucleosome interaction strength under physiological conditions [165]. The residue‐level CG model resolves ions explicitly and captures key electrostatic interactions among nucleosomes. Simulations estimate the binding free energy between two nucleosomes to be approximately $9\;k_{B}T$ under physiological salt and ion conditions, supporting the view that intrinsic physicochemical forces are substantial contributors to chromatin phase behavior and higher‐order organization. (Adapted from Lin and Zhang, *eLife* 12, e90073, 2024 [165]; licensed under CC BY 4.0).

The structural and functional integrity of chromatin begins at the nucleosome, the core unit formed by DNA wrapped around a histone octamer, establishing the scaffold for higher‐order genome organization [120, 162, 163]. A crucial contributor to chromatin architecture is the intrinsic physicochemical properties of nucleosomes, particularly via electrostatic interactions mediated by the highly charged DNA, histone cores, and their intrinsically disordered N‐terminal tails [15, 164]. Recent high‐resolution modeling further underscores this: an explicit‐ion all‐atom model suggested an effective nucleosome–nucleosome attraction on the order of several $k_{B}T$ under physiological conditions (Figure 11), a finding remarkably consistent across ionic environments and reinforcing the importance of these intrinsic forces in driving chromatin condensation and phase behavior [165]. Complementing these findings, systematic bottom‐up coarse‐grained models with explicit ions have accurately captured nucleosome–nucleosome attractions mediated by multivalent cations [166], and further revealed how salt‐dependent interactions dictate the conformational variability of nucleosome arrays [167]. This intrinsic compaction is further enhanced by architectural chromatin proteins, such as linker histones (e.g., H1), which bind at DNA entry/exit sites and stabilize internucleosomal stacking to promote further fiber compaction [132, 168].

**FIGURE 12:**
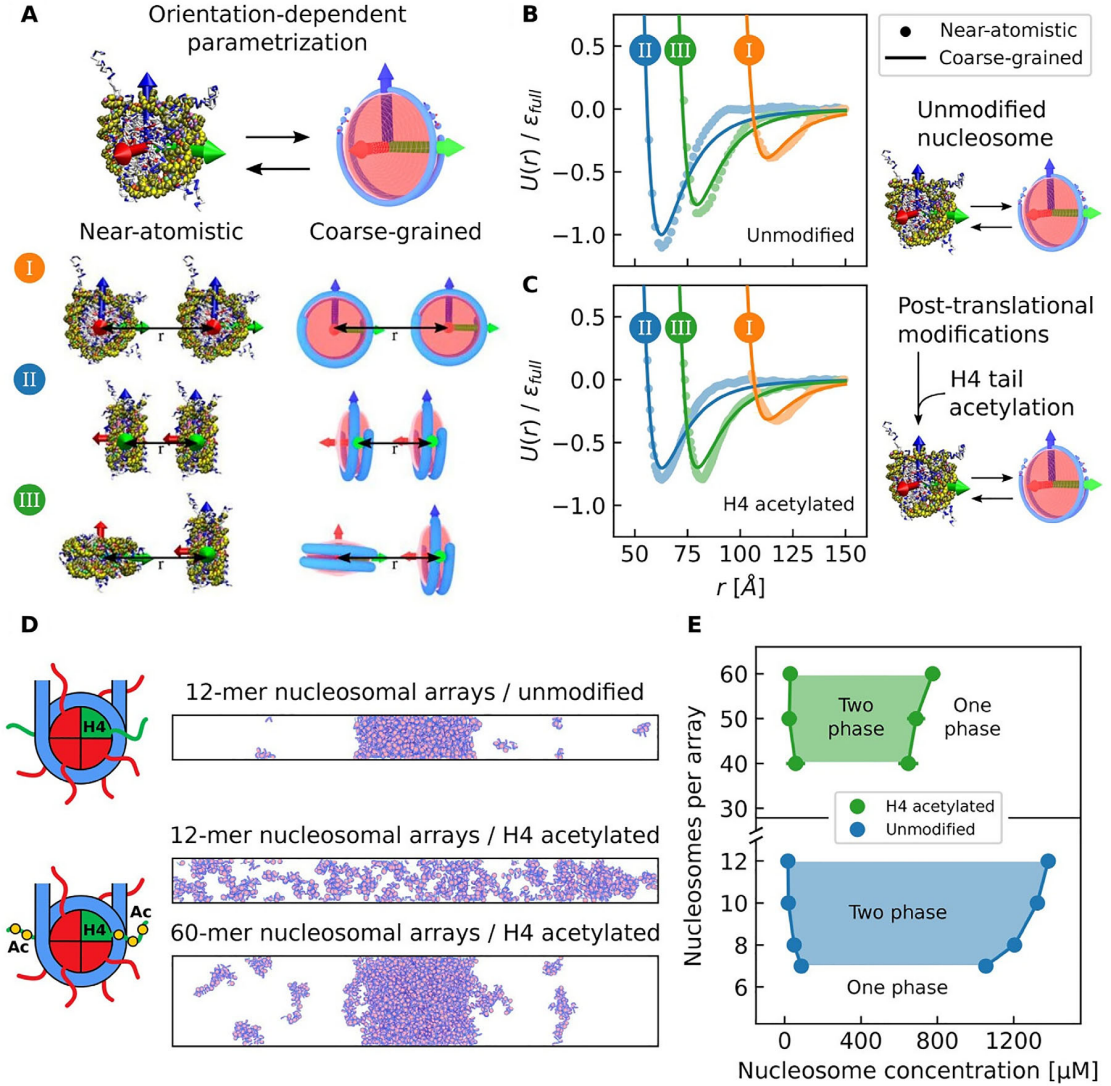
CG modeling of how histone post‐translational modifications regulate chromatin condensate properties [169]. (A) The nucleosome is represented as a rigid, non‐spherical CG unit with six orientational degrees of freedom, enabling accurate modeling of orientation‐dependent internucleosomal interactions. (B,C) These interactions are parameterized using data from high‐resolution simulations to capture the effects of modifications such as H4‐tail acetylation on the CG potential. (D,E) Subsequently, simulations of nucleosomal arrays demonstrate that while H4‐tail acetylation induces decompaction in short (12‐mer) arrays, it still permits phase separation in longer (60‐mer) arrays. This reveals that acetylation acts as a fine‐tuning mechanism for condensate formation and material properties, modulating chromatin's phase behavior in a length‐dependent manner. (Adapted from Golembeski and Lequieu, *J. Phys. Chem. B* 128, 10593‐10603, 2024 [169]; licensed under CC BY 4.0).

The phase behavior of nucleosomes is dynamically regulated by histone post‐translational modifications (PTMs). These chemical marks act as a tunable “code” that modulates histone tail interactions, for example, phosphorylation can weaken nucleosome‐DNA affinity, while acetylation can dissolve chromatin droplets [9]. Such modifications enable selective formation and segregation of chromatin domains. Computational models provide key mechanistic insights: Golembeski et al. demonstrated via CG simulations that H4‐tail acetylation causes pronounced decompaction in short nucleosomal arrays but preserves phase separation in longer arrays (Figure 12), highlighting that PTMs fine‐tune, rather than switch off, condensate behavior [169]. In parallel, modeling of nucleosome plasticity, corresponding to the spontaneous “breathing” motions where DNA transiently unwraps from the histone core, revealed that this conformational flexibility enhances the transient nature and heterogeneity of nucleosome‐nucleosome contacts, increasing effective multivalency and promoting liquid‐like chromatin condensates [163].

**FIGURE 13:**
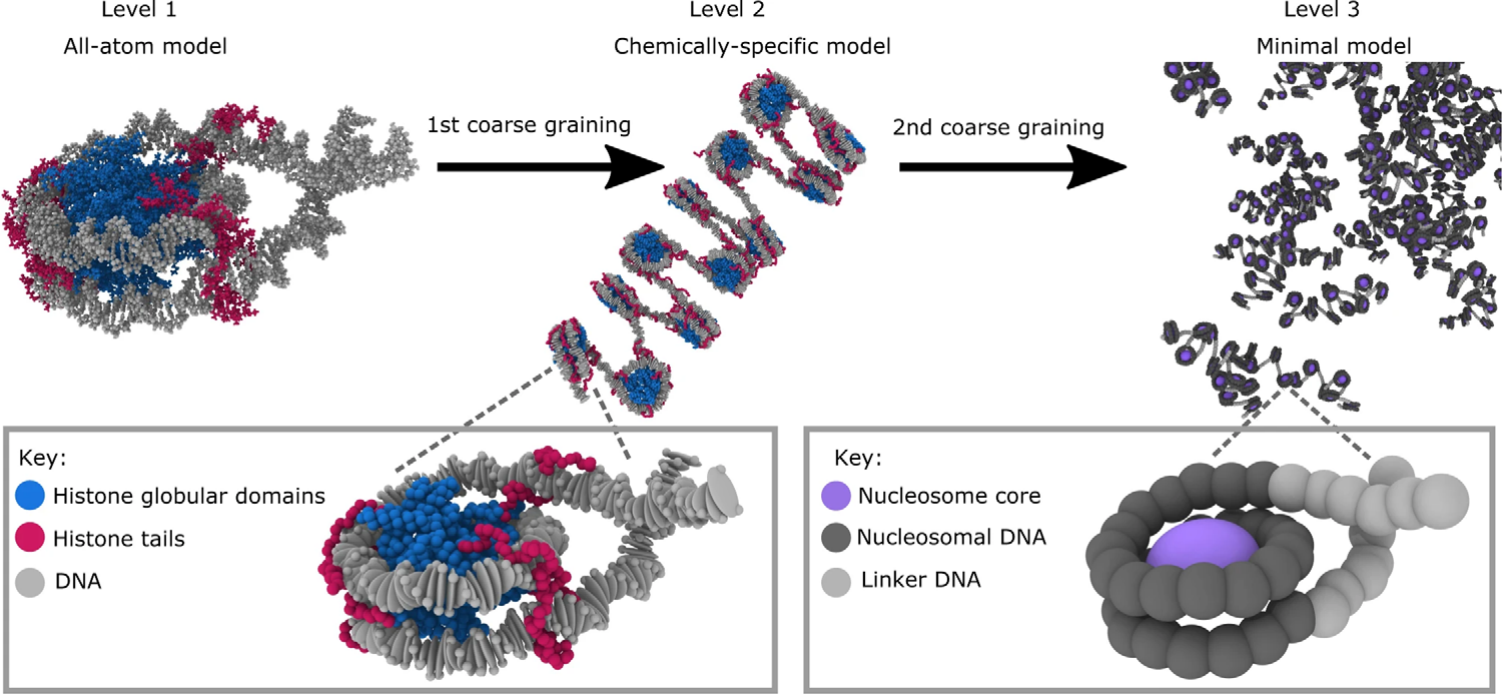
Multiscale modeling framework for chromatin phase separation developed by Farr et al. [163]. This approach integrates three hierarchical resolution levels: **Level 1**: High‐resolution AA MD of nucleosomes and DNA, providing detailed physicochemical parameters. **Level 2**: Chemically specific CG model, using residue‐ and base‐pair‐level representations to bridge atomistic details with mesoscale chromatin behavior. **Level 3**: Minimal CG model, enabling simulation of large chromatin systems (thousands of nucleosomes) and their phase behavior. This multiscale strategy reveals that nucleosome plasticity, which is manifested as thermal breathing and conformational variability, enhances internucleosomal multivalency, supporting both liquid‐like chromatin folding and LLPS. Importantly, such dynamic plasticity appears pivotal for chromatin's liquid‐like behavior under physiological conditions. (Adapted from Farr et al., *Nat. Commun*. 12, 2883, 2021 [163]; licensed under CC BY 4.0).

Elucidating the interplay of multi‐scale determinants of chromatin organization necessitates a sophisticated bottom‐up computational approach. Farr et al. exemplify this with their multi‐scale modeling framework (Figure 13), which hierarchically integrates: (i) high‐resolution AA MD simulations capturing nucleosome physicochemistry, (ii) a chemically specific CG model parameterized at the residue and base‐pair level, and (iii) a minimal CG model enabling simulations of thousands of nucleosomes [163]. This framework revealed that constraining intrinsic nucleosome plasticity disrupts LLPS behavior, thereby establishing a causal link between dynamic nucleosome “breathing” and liquid‐like chromatin condensation. Such rapidly developing multiscale strategies, implemented using advanced MD engines like LAMMPS or GPU‐accelerated platforms such as HOOMD‐blue, have become mainstream in chromatin biophysics. They enable seamless exploration from atomistic mechanisms (e.g., ion‐mediated interactions) to large‐scale, higher‐order chromatin dynamics and condensate formation [26, 164, 169].

**FIGURE 14:**
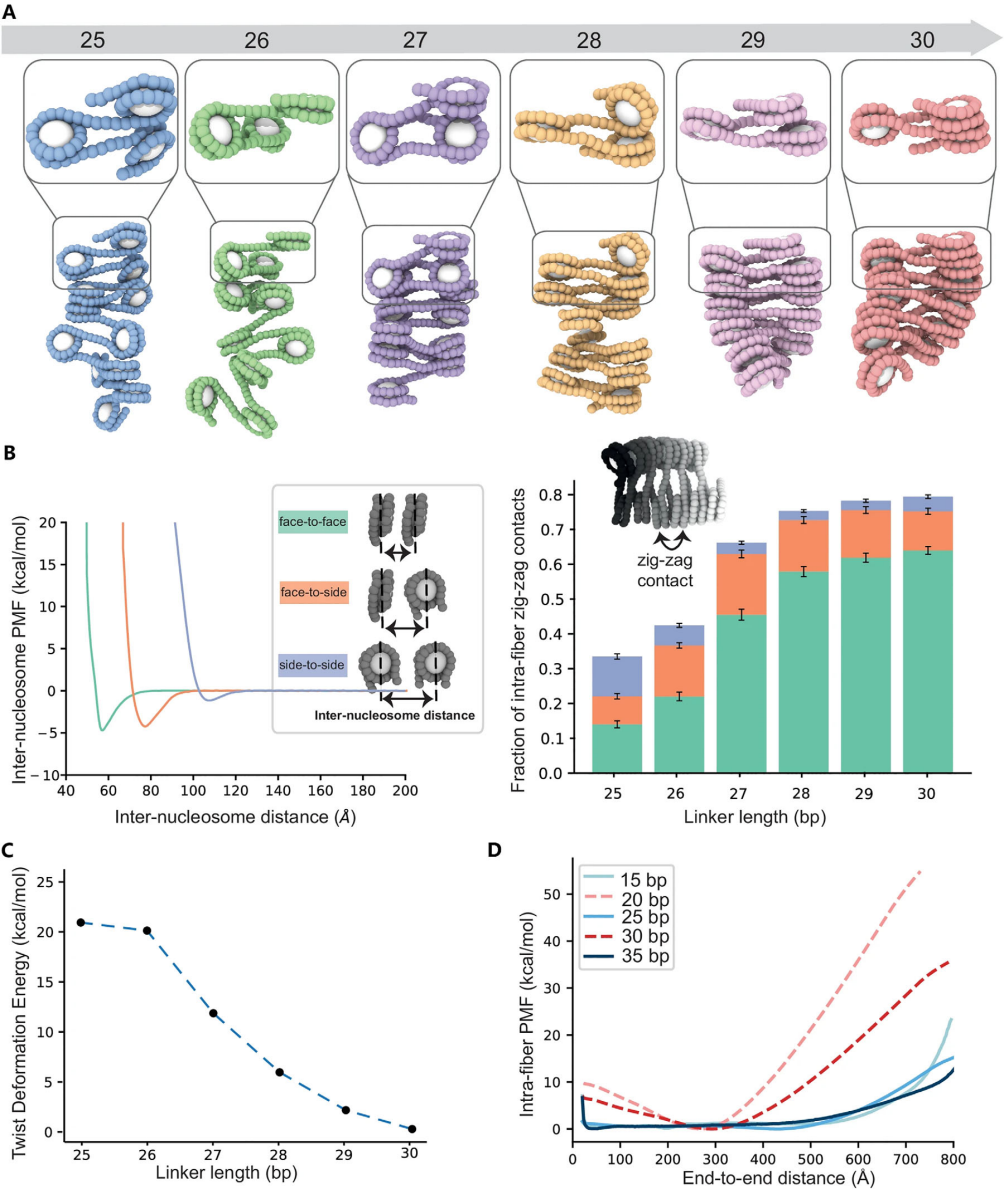
Fine‐tuning of higher‐order chromatin assembly by nucleosome spacing [170]. Chromatin arrays with DNA linkers from 25 bp to 30 bp were examined through a combination of in vitro assays and MD simulations. As DNA‐linker length increases, condensates become progressively less thermodynamically stable and display higher internal nucleosome mobility, reflecting a critical balance between inter‐fiber stacking (promoting phase separation) and intra‐fiber stacking (inhibiting it). This study also demonstrates that chromatin remodelers can modulate condensate behavior by adjusting nucleosome positioning to favor or disfavor stacking configurations, thereby epigenetically regulating chromatin compaction and dynamics. (Adapted from Chen et al., *Nat. Commun*. 16, 6315, 2025 [170]; licensed under CC BY 4.0).

These simulation studies collectively reveal several fundamental physical principles underlying chromatin organization. A consistent finding is that nucleosome condensates are structurally disordered, challenging the classic, regular 30‐nm fiber model [169]. The DNA‐linker length critically modulates condensate formation: arrays with 10 N + 5 bp linkers phase‐separate more readily than those with 10 N bp, due to a balance between inter‐fiber stacking (which promotes condensation) and intra‐fiber interactions (which inhibit it), as demonstrated by the recent experimental and simulation evidence showing oscillatory phase‐separation thresholds with single‐base resolution (Figure 14) [170]. In alignment with this, recent structural characterizations combining cryo‐electron tomography and molecular dynamics simulations have demonstrated that internucleosomal DNA linker length directly controls nucleosome arrangement and histone tail interactions. This structural modulation balances intra‐ and intermolecular interactions, thereby governing the molecular network and macroscopic material properties of chromatin condensates [171]. Leveraging near‐atomistic coarse‐grained frameworks such as OpenCGChromatin, researchers can now simulate large‐scale chromatin condensates to capture how these sequence‐level physical principles dictate emergent phase separation [172]. Furthermore, simulations have confirmed that HP1‐mediated bridging of H3K9me3‐marked histones is a core mechanism for heterochromatin formation [81], and that different multivalent cations induce distinct packing structures by binding to specific sites on the nucleosome [164]. Finally, models like RouseTIC suggest that chromatin can exist in a poised, near‐critical state, where minor regulatory perturbations can yield large‐scale structural transitions, underscoring its dynamic responsiveness [173]. Together, these computational investigations provide a quantitative bridge from molecular‐scale interactions to emergent higher‐order chromatin structure and function.

Ultimately, these multiscale modeling efforts—from isolated nucleosomes and polynucleosome arrays up to kilobase‐scale genes—provide a comprehensive view of how protein binding and local physicochemical interactions regulate genome folding [174, 175]. At this microscopic level, the intrinsic phase‐separation capacity fundamentally challenges the classical dichotomy of an “open” euchromatin versus a “closed” heterochromatin. Recent advanced imaging and single‐nucleosome tracking experiments have revealed that both euchromatin and heterochromatin exist primarily as condensed domains, but critically differ in their material properties: euchromatin behaves as a dynamic, liquid‐like condensate that allows penetration of small transcription factors, whereas heterochromatin adopts a more constrained, gel‐like state [176, 177]. Integrating computational models with these evolving experimental views is thus essential for decoding the physical basis of DNA accessibility and gene regulation.

Chromatin‐associated proteins are central architects of functional condensates, acting at multiple levels to shape nuclear organization. Heterochromatin proteins, such as HP1α, bind H3K9me3‐marked nucleosomes and drive genome compartmentalization via multivalent interactions, contributing to micro‐phase separation in pericentromeric heterochromatin and B‐type chromatin domains [178, 179, 180]. RNA‐binding proteins such as FUS and nucleophosmin engage in RNA‐mediated phase separation to assemble membraneless substructures such as nucleoli and DNA damage‐response hubs [181, 182]. At the biological level, these protein‐chromatin condensates do more than merely package DNA; they create highly specialized biochemical microenvironments [183]. For example, phase‐separated DNA damage‐response hubs locally concentrate repair factors while physically excluding exonucleases, thereby preserving genome integrity [184]. Similarly, the liquid‐to‐gel transitions of HP1‐mediated condensates effectively silence transcription by mechanically restricting the accessibility of RNA polymerase and transcription machinery, ensuring precise spatiotemporal control of gene expression [185, 186]. Transcriptional regulators, most notably BRD4 and MED1, phase‐separate at super‐enhancers, concentrating coactivators, and RNA Pol II into dynamic condensates that enhance transcription initiation and looping interactions [150, 187].

**FIGURE 15:**
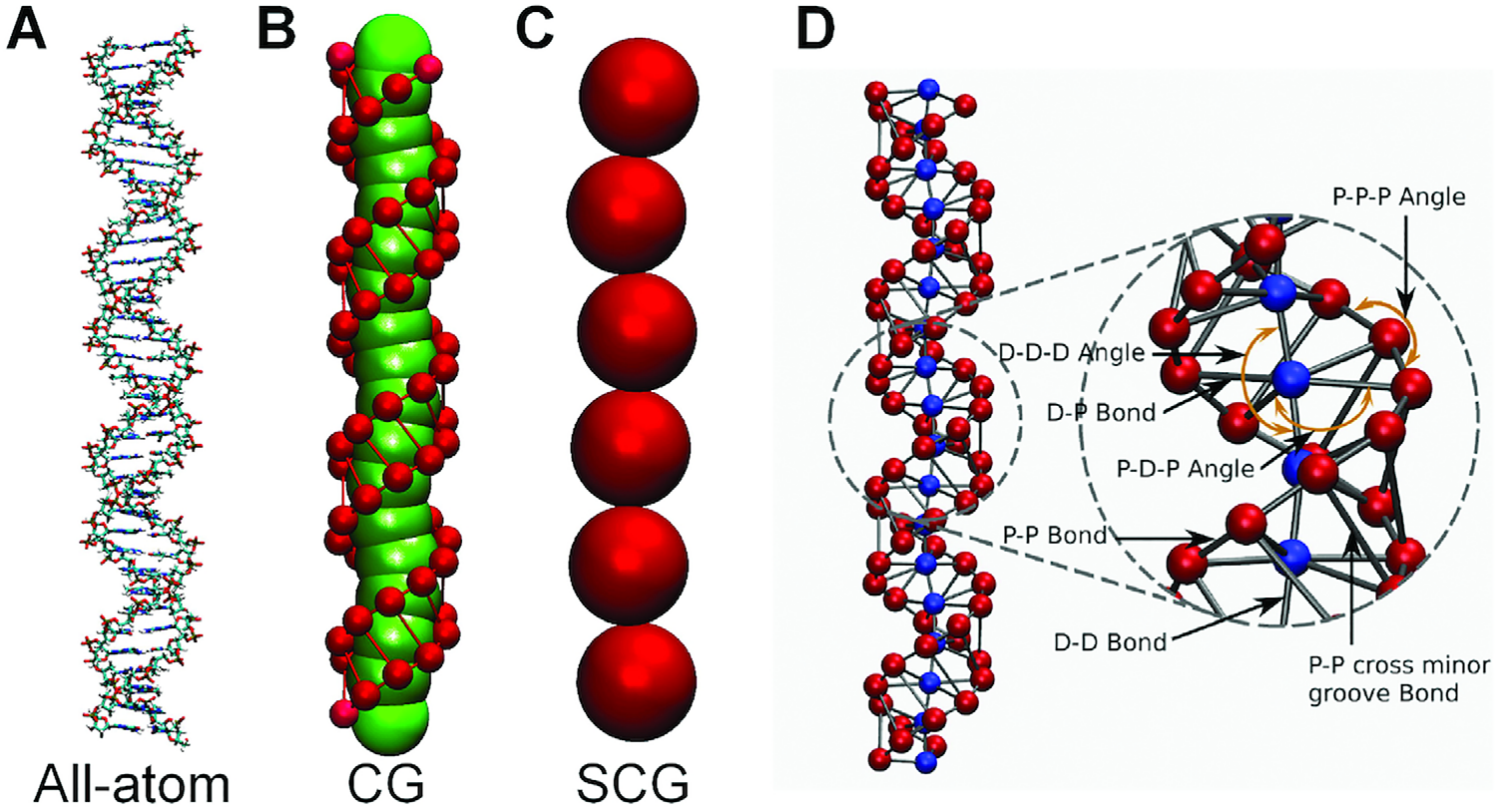
Hierarchical multiscale coarse‐graining of DNA phase separation as developed by Sun et al. [162]. The modeling pipeline progresses systematically across three resolution scales: (A) AA representation of DNA captures full atomic interactions and used to derive effective interaction potentials. (B) First‐level CG model simplifies the DNA double helix into dual‐bead units that preserve structural helicity and inter‐DNA interactions. (C) Super CG (SCG) “beads‐on‐a‐string” model collapses DNA into single‐bead units to enable simulations of large DNA constructs (e.g., ∼10 kb), effectively capturing mesoscale phase behavior such as toroidal or fibrous aggregates with hexagonal packing. (D) Detailed schematic of the CG model's internal potentials and geometry display bond interactions, backbone and minor‐groove cross‐link interactions and angle potentials, which are essential to preserving DNA structural fidelity in CG model. This parameter‐free, bottom‐up approach ensures physical consistency across scales and reproduces experimentally observed DNA condensation morphologies, including liquid crystalline and toroidal phases, demonstrating its robustness in simulating DNA phase transitions [162]. (Adapted from Sun et al., *Nucleic Acids Res*. 47, 5550‐5562, 2019 [162]; licensed under CC BY 4.0).

Computational modeling has proven indispensable for unraveling the molecular grammar that governs chromatin organization on multiple scales. Even DNA, the foundational substrate, reveals rich behavior when probed through multiscale simulations. A notable example is the hierarchical modeling framework introduced by Sun et al. (Figure 15), which spans atomic to mesoscale representations. Starting from AA MD simulations, progressing through chemically specific CG models, and culminating in super CG (SCG) “beads‐on‐a‐string” constructs, this bottom‐up approach demonstrated that short DNA molecules, in the presence of multivalent ions, self‐assemble into liquid crystalline phases, providing a foundational baseline for DNA compaction [162]. Building on such DNA‐centric frameworks, CG polymer models have been extended to study specific protein‐DNA systems. For instance, Su et al. developed a CG model of DNA and its sensor protein cGAS, illustrating that condensate formation involves a multi‐step process critically dependent on both cGAS dimerization and protein‐DNA interactions. This highlights how multivalency and intrinsic disorder orchestrate assembly dynamics [25].

**FIGURE 16:**
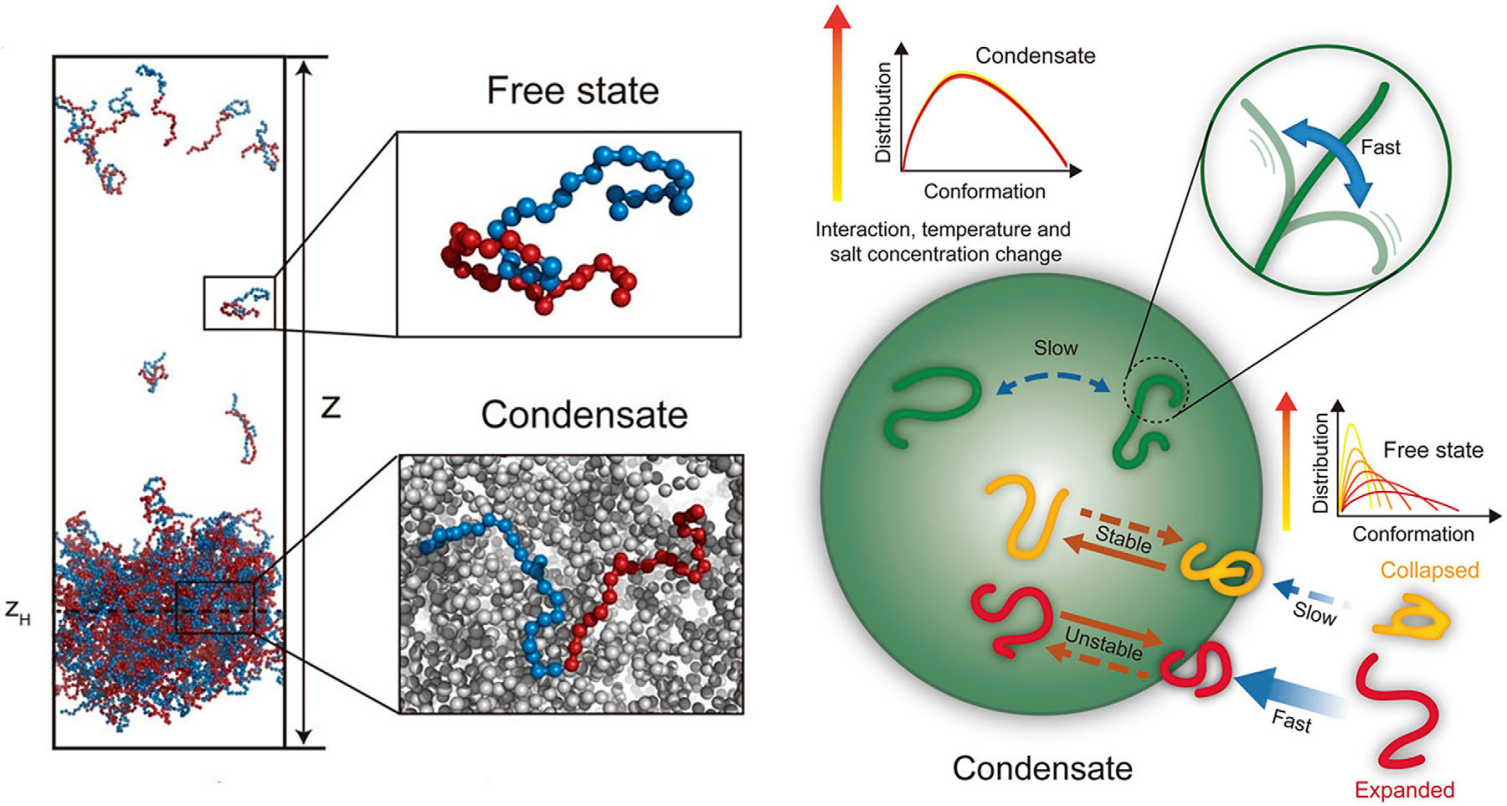
CG model elucidating how IDP phase separation is governed by a balance between thermodynamic stability and kinetic accessibility [153]. The model tracks the transition of IDP chains from dilute to dense condensate states under varying interaction strengths, ionic strengths, and temperatures. Key insights include: (i) conformational characteristics of IDPs in the dense phase remain conserved and more extended than in the dilute phase; (ii) local chain flexibility persists despite slowed global dynamics due to condensate viscosity; and (iii) condensate formation kinetics exhibit a non‐monotonic dependency on interaction strength, where stronger interactions enhance stability but can slow assembly. These findings underscore that thermodynamic stability and kinetic accessibility are decoupled and optimized via a “speed‐stability” trade‐off in phase separation. (Adapted from Zhang and Chu, *J. Chem. Phys*. 161, 095102, 2024 [153]; licensed under CC BY 4.0).

The behavior of IDPs presents a unique challenge due to their complex, sequence‐dependent dynamics. Representative simplified CG models, such as that shown in Figure 16, offer foundational insights, demonstrating that the kinetics of condensate assembly can be decoupled from thermodynamic stability: the strongest intermolecular interactions do not necessarily yield the fastest assembly dynamics [153]. To capture sequence‐specific behaviors in proteins containing both folded and disordered domains, more sophisticated force fields are essential. Equally critical is the accurate representation of electrostatic forces in charge‐rich biomolecular condensates containing nucleic acids and intrinsically disordered proteins. To this end, recent coarse‐grained models such as Mpipi‐Recharged have introduced chemically informed, pair‐specific electrostatic potentials that capture the intricate effects of charge blockiness, complex coacervation, and salt modulation without requiring explicit solvation [188]. Complementing such advances, the MOFF force field, developed by Latham and Zhang, represents a major step forward in unified protein modeling [179]. By combining energy landscape theory with maximum‐entropy parameterization, it achieves consistent accuracy for both folded proteins and IDPs. Applied to HP1 homologs, MOFF successfully resolved subtle structural distinctions between highly similar variants and uncovered the multivalent interactions that stabilize their higher‐order condensate assemblies. These results highlight MOFF as a powerful near‐atomistic tool for investigating LLPS separation with sequence‐level specificity.

**FIGURE 17:**
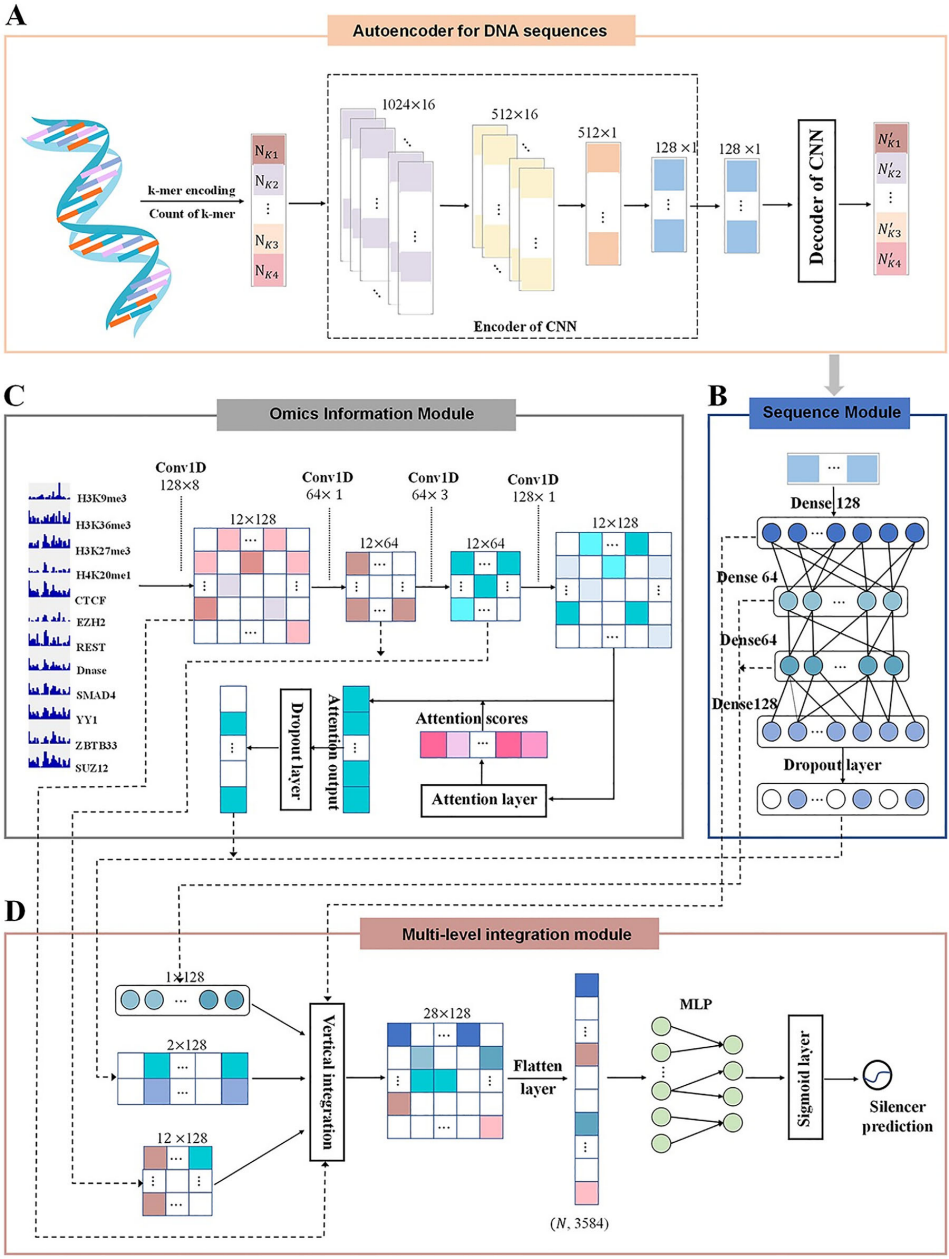
Deep learning framework for predicting cell‐specific silencers [55]. DeepICSH, developed by Zhang et al., integrates raw DNA sequence data with multi‐omics signals, such as chromatin accessibility and histone modifications, through a multi‐level convolutional neural network enhanced with attention mechanisms and skip connections. This architecture automatically learns biologically relevant feature combinations and effectively distinguishes strong and weak silencers across cell types. The model provides interpretable predictions via attention‐based visualization with highlighting sequence motifs and epigenetic patterns underlying repressive activity. (Adapted from Zhang et al., *Brief Bioinform*. 24, bbad316, 2023 [55]; licensed under CC BY‐NC 4.0).

Complementing physics‐based simulations is a robust ecosystem of data‐driven approaches for the sequence‐level analysis of phase separation. Resources like PhaSepDB curate experimentally verified phase‐separating proteins, annotating them with sequence features, droplet characteristics, and regulatory information [56]. Machine learning predictors, such as ESPredictor [189], PSPHunter [190], Metapredict [191], PSAP [192], FuzDrop [193], IUPred [194], and ParSe [195], use sequence composition and biophysical features (e.g., disorder propensity, low‐complexity regions, hydrophobicity) to forecast LLPS‐driving proteins and droplet‐promoting domains. The sophistication of these computational tools continues to advance with deep‐learning frameworks that integrate diverse biological datasets. For instance, DeepICSH by Zhang et al. combines DNA sequence with epigenomic data to predict cell‐type–specific silencers (Figure 17), illustrating how neural networks can decode complex regulatory logic from genomic inputs [55]. A recent breakthrough that bridges the simulation and data‐driven methods comes from Bülow et al., who used CG MD simulations to train a machine learning model that accurately predicts the saturation concentration for phase separation directly from any disordered sequence, showcasing the future of integrated computational approaches [58]. This model was validated on proteome‐wide IDRs, capturing sequence‐to‐phase behavior relationships and offering a powerful tool for interpreting, designing, and simulating biomolecular condensates.

## 6 Model Validation, Identifiability, and Uncertainty

As computational models of 3D genome organization grow in sophistication, a critical challenge arises: distinguishing between models that are genuinely constrained by experimental data and those that are merely compatible with it. Because distinct physical mechanisms can sometimes yield similar macroscopic phenotypes (e.g., checkerboard contact patterns or clustered domains), researchers face the pitfall of model degeneracy and poor parameter identifiability [64]. A model is considered identifiable only if the available data uniquely determine its parameter values and underlying physical mechanisms. Therefore, robust uncertainty quantification and strategic model validation are essential to elevate computational frameworks from descriptive tools to predictive theories.

To overcome identifiability issues, it is crucial to understand exactly, which experimental observables constrain which physical parameters. Bulk Hi‐C provides robust, population‐averaged contact probabilities, making it an excellent resource for defining effective interaction affinities, compartmental segregation, and topological domain boundaries [83]. However, bulk Hi‐C alone is inherently degenerate: it cannot easily differentiate between stable, long‐lived contacts and transient, frequent ones, nor does it yield absolute physical distances. Single‐cell Hi‐C and multiplexed chromatin tracing bridge this gap by capturing structural heterogeneity, cooperative (multi‐way) interactions, and the precise variance of 3D conformations across a cell population [19, 132]. Meanwhile, fluorescence imaging (e.g., FISH) provides absolute spatial distances and volume constraints, which are indispensable for calibrating polymer stiffness and density [196]. Finally, live‐cell imaging of dynamics (e.g., FRAP and tracking mean squared displacement, MSD) uniquely constrains the timescales, viscosities, and binding/unbinding kinetics of phase‐separating components—parameters that static structural data leave entirely unconstrained [110, 153].

Best practices in the field increasingly demand rigorous cross‐modality validation. A robust model should be parameterized on one data modality and evaluated on an independent one; for instance, deriving a coarse‐grained polymer potential strictly from Hi‐C or epigenetic data, and subsequently demonstrating that the model accurately predicts FISH spatial distance distributions or imaging‐based compartmentalization without additional fitting [83, 120].

At the microscopic and molecular scales, the rigorous validation of CG representations and their underlying force fields are equally critical, as the choice of resolution and parameterization scheme can significantly skew predicted phase behaviors. For bottom‐up CG models derived from all‐atom simulations, robust validation requires demonstrating that the simplified model faithfully reproduces the structural ensembles (e.g., radius of gyration, radial distribution functions) and thermodynamic properties (e.g., binding free energies) of the explicit high‐resolution reference. Furthermore, sequence‐specific protein force fields must be rigorously benchmarked against in vitro experimental observables—such as single‐molecule FRET, small‐angle X‐ray scattering (SAXS), and nuclear magnetic resonance (NMR) data—to confirm they accurately capture the conformational flexibility and dimensions of IDRs. Crucially, a reliable force field for biomolecular condensates must be validated by its ability to predict experimental phase diagrams, specifically matching saturation concentrations ($C_{sat}$) and condition‐dependent phase boundaries (binodals), rather than merely driving non‐specific aggregation. Ensuring this thermodynamic consistency across resolutions is paramount to guarantee that emergent mesoscale condensates are grounded in authentic molecular physics.

The ultimate test of a model's predictive power and mechanistic validity, however, lies in its ability to forecast the outcomes of targeted in silico perturbations. A truly constrained model must accurately recapitulate architectural shifts following experimental disruptions. Essential perturbation benchmarks include: (i) structural changes upon loop extrusion loss (e.g., predicting the disappearance of TADs and strengthening of compartments upon cohesin or CTCF depletion) [116]; (ii) heterochromatin decompaction following HP1 depletion or mutational disruption of its IDRs [81]; (iii) remodeling of domains following precise editing of post‐translational modifications (PTMs) [169]; and (iv) the dissolution or altered dynamics of transcriptional condensates upon chemical inhibition of transcription (e.g., via Actinomycin D or DRB) [157]. Testing models against such diverse perturbations ensures that the inferred mechanisms reflect true biological causality rather than mathematical overfitting.

To assist researchers in navigating these challenges, we summarize a brief “validation checklist” (Box 2), offering a prescriptive guide to evaluating computational models of genome architecture.

**Box 2. A Prescriptive Checklist for Chromatin Model Validation**.

To rigorously establish that a computational model is constrained by data rather than merely compatible with it, modelers should map their specific modeling objectives to orthogonal validation data and targeted perturbations. This table provides a practical guide for overcoming parameter identifiability issues.

**Table:** 

**Modeling Objective**	**Key Parameters**	**Primary Data (For Parameter Fitting)**	**Orthogonal Validation (For Identifiability)**	**Mechanistic Perturbation (In Silico vs. In Vivo)**	**References**
**1. Static 3D Organization (Compartments & TADs)**	Interaction affinities, polymer stiffness, extruder processivity.	Bulk Hi‐C (contact frequencies) ChIP‐seq / Epigenetic marks (1D features)	**Multiplexed FISH**: Validates absolute physical distances (nm). **Single‐cell Hi‐C**: Validates structural heterogeneity and multi‐way contacts.	Depletion of loop extruders (e.g., cohesin/CTCF degrons). Deletion of specific boundary elements.	[197, 198, 199]
**2. Heterochromatin Condensation & Valency**	Multivalent binding strength, stoichiometry, density.	ChIP‐seq (binder localization) In vitro phase diagrams (saturation concentrations).	**Live‐cell imaging**: Validates number of condensates and cluster size distributions in the nucleus.	HP1 depletion. Editing of Post‐Translational Modifications (e.g., targeted acetylation/methylation).	[200, 201, 202]
**3. Condensate Kinetics & Material Properties**	Binding/unbinding rates ($k_{\mathrm{on}}/k_{\mathrm{off}}$), viscosity, surface tension.	FRAP (fluorescence recovery curves). Single‐particle tracking (MSD).	**Droplet fusion kinetics**: Validates viscocapillary rounding times. **Rheology assays**: Validates elastic vs. viscous moduli.	Mutating IDRs to alter sticker/spacer valency. 1,6‐HD treatment.	[41, 43, 202]
**4. Active/Transcriptional Condensate Hubs**	Motor activity, RNA synthesis rates, feedback thresholds.	RNA‐seq / GRO‐seq (transcriptional output). ATAC‐seq (accessibility).	**Nascent RNA FISH**: Validates spatial co‐localization of active loci. **Non‐equilibrium tracking**: Entropy production / detailed balance violation.	Transcription inhibition (e.g., Actinomycin D, DRB). ATP depletion (shutting off active mechanics).	[203, 204]

**Key Principles for Identifiability**:

- **Avoid Circularity**: Never validate the model using the same modality used to train it (e.g., fitting affinities to Hi‐C and validating by simulating a Hi‐C map).
- **Resolve Degeneracy**: Bulk data averages out dynamics. A model capturing a static “checkerboard” Hi‐C pattern is degenerate; dynamic data (FRAP, single‐cell variance) is required to identify if the pattern arises from stable cross‐links versus highly dynamic liquid droplets.

## 7 Summary and Outlook

This review highlights the indispensable roles of multiscale computational modeling in elucidating how phase separation orchestrates hierarchical genome organization and transcriptional control. We show how physics‐based models, such as loop‐extrusion and polymer phase‐separation frameworks, provide mechanistic insight across scales, from A/B compartments and TADs to transcriptional condensates [36, 116]. Concurrently, data‐driven models, informed by sequence and epigenomic features, allow accurate prediction of population‐level 3D chromatin structure and capture cell‐to‐cell heterogeneity with single‐cell resolution [84, 205]. A key unifying insight is the dynamic balance between passive phase separation that drives compartmental segregation and active processes such as loop extrusion that mix chromatin and modulate structural organization. These models further reveal that genome folding is finely tuned by epigenetic modifications, intrinsic DNA sequence patterns, chromatin‐binding proteins, and nuclear architectural constraints (e.g., lamina association) [3, 106, 109].

Looking forward, a key priority is to move beyond quasi‐equilibrium pictures and explicitly encode non‐equilibrium drivers within active‐matter frameworks. To construct a truly predictive “active 4D nucleome,” future multiscale models must integrate a concrete roadmap of canonical active modules. We propose a schematic “minimal active model stack” (Box 3) that explicitly couples four core non‐equilibrium components: (i) loop extrusion as a continuous active mixing term that counteracts passive phase separation [121, 122, 123]; (ii) transcription as localized, time‐dependent source and sink terms that drive RNA‐mediated re‐entrant phase transitions [52, 204, 206]; (iii) enzymatic writer/eraser kinetics coupled with 3D spatial feedback to model epigenetic memory [39, 81, 143]; and (iv) mechanical heterogeneity, treating chromatin as a viscoelastic field that constrains condensate growth and transport [60, 207, 208, 209, 210]. Unifying these modules will clarify which open questions in genome architecture are fundamentally governed by thermodynamic driving forces, kinetic barriers, or mechanical constraints. A powerful proof‐of‐concept for such holistic integration was recently demonstrated in a complete 4D spatial and kinetic model of the minimal bacterium JCVI‐syn3A [211]. By coupling SMC‐driven chromosome segregation via Brownian dynamics with metabolic dNTP fluxes and morphological constraints, this hybrid simulation successfully captured both population‐level measurements and single‐cell stochasticity. Extending similar whole‐cell, multi‐physics frameworks to the highly compartmentalized eukaryotic nucleome represents a critical next frontier.

**Box 3. A Minimal Active Model Stack for the 4D Nucleome**.

To transition from quasi‐equilibrium descriptions to fully predictive non‐equilibrium frameworks, future computational models should integrate these four canonical active modules. This stack clarifies how specific physical implementations map to biological phenomena and fundamental physical principles.

**Table:** 

**Active Module**	**Computational Implementation**	**Biological Phenomena**	**Primary Physics**	**References**
**1. Active Mixing** (Loop Extrusion)	1D driven motor stepping coupled to 3D polymer constraints. Active noise or non‐conservative force terms ($F_{\mathrm{act}}$).	TAD formation and boundaries. Antagonism to passive compartmental segregation (mixing vs. demixing).	**Kinetic** (processivity, stall rates) & **Mechanical** (motor stall force).	[121, 122, 123]
**2. Non‐Equil. Fluxes** (Transcription & RNA)	Localized, time‐dependent source/sink terms (production/degradation). Concentration‐dependent phase envelopes.	Transcriptional bursting. Condensate repositioning. Re‐entrant phase behavior (dissolution via RNA excess).	**Thermodynamic** (phase boundaries) & **Kinetic** (production/decay rates).	[52, 204, 206]
**3. 3D Epigenetic Feedback** (Writer/Eraser)	Stochastic state transitions (e.g., Markov recoloring) linked to 3D proximity. Auto‐catalytic feedback loops.	Spreading of heterochromatin. Epigenetic memory across cell cycles. Switch‐like domain formation.	**Kinetic** (enzymatic rates) & **Thermodynamic** (bistability/memory).	[39, 81, 143]
**4. Viscoelastic Field** (Mechanics)	Spatially varying elastic networks or poroelastic medium models. Local stiffness/pressure fields coupled to concentration.	“Elastic ripening” and arrested droplet fusion. Anomalous subdiffusion of loci. Shape deformation of condensates.	**Mechanical** (stiffness, elasticity) & **Kinetic** (relaxation times).	[60, 207, 208, 209, 210]

**How modules couple in future frameworks**:

In a fully integrated model, **Module 3** (epigenetics) establishes the initial interaction landscape, guiding **Module 1** (extrusion), which dynamically reshapes 3D proximity. This folding triggers **Module 2** (transcription), injecting RNA fluxes that form local condensates. Finally, **Module 4** (mechanics) dictates the physical limits of how large these condensates can grow and how fast the chromatin network relaxes around them.

We anticipate next‐generation multiscale models that couple atomistic fidelity (e.g., PTMs, ion specificity) to chromosome‐wide dynamics via transferable CG representations and learned force fields; differentiable and machine‐learning potentials can absorb heterogeneous biophysical data while retaining physical priors [212, 213, 214]. On the data side, rapidly developing single‐cell and imaging experimental techniques, including high‐throughput droplet single‐cell Hi‐C and multiome assays, chromatin tracing with nanometre‐scale DNA mapping, and pooled CRISPR screens read out in situ, will provide rich constraints for model training, validation, and causal inference [215, 216, 217, 218, 219, 220]. In parallel, sequence‐to‐phase frameworks now predict condensate thermodynamics and saturation concentrations directly from disordered sequences, enabling proteome‐scale hypothesis generation and variant effect mapping [58, 190, 221, 222]. Achieving a predictive 4D nucleome will require open benchmarks, uncertainty quantification, and systematic perturbations that integrate sequencing, imaging, and simulation. We anticipate that success will deepen mechanistic understanding of gene regulation and illuminate how genetic variation reshapes nuclear biophysics in disease, informing strategies that target condensate and chromatin material properties [223, 224, 225].

## Conflicts of Interest

The authors declare no conflicts to disclose.

## Data Availability

Data sharing is not applicable to this article as no new data were created or analyzed in this study.
